# A geospatial platform for the tectonic interpretation of low-temperature thermochronology Big Data

**DOI:** 10.1038/s41598-023-35776-3

**Published:** 2023-05-26

**Authors:** Samuel C. Boone, Fabian Kohlmann, Wayne Noble, Moritz Theile, Romain Beucher, Barry Kohn, Stijn Glorie, Martin Danišík, Renjie Zhou, Malcolm McMillan, Angus Nixon, Andrew Gleadow, Xiaodong Qin, Dietmar Müller, Brent McInnes

**Affiliations:** 1 School of Geography, Earth and Atmospheric Sciences, The University of Melbourne, Melbourne, VIC 3010 Australia; 2 Department of Earth Sciences, University of Adelaide, Adelaide, SA 5005 Australia; 3 Lithodat Pty Ltd, Melbourne, VIC 3030 Australia; 4 Research School of Earth Sciences, Australian National University, Canberra, Australia; 5 John de Laeter Centre, Curtin University, Bentley, WA 6102 Australia; 6 School of Earth and Environmental Sciences, University of Queensland, Brisbane, Australia; 7 EarthByte Group, School of Geosciences, University of Sydney, Sydney, NSW 2006 Australia

**Keywords:** Tectonics, Geochemistry

## Abstract

Low-temperature thermochronology is a powerful tool for constraining the thermal evolution of rocks and minerals in relation to a breadth of tectonic, geodynamic, landscape evolution, and natural resource formation processes through deep time. However, complexities inherent to these analytical techniques can make interpreting the significance of results challenging, requiring them to be placed in their geological context in 4-dimensions (3D + time). We present a novel tool for the geospatial archival, analysis and dissemination of fission-track and (U-Th)/He data, built as an extension to the open-access *AusGeochem* platform (https://ausgeochem.auscope.org.au) and freely accessible to scientists from around the world. To demonstrate the power of the platform, three regional datasets from Kenya, Australia and the Red Sea are placed in their 4D geological, geochemical, and geographic contexts, revealing insights into the tectono-thermal evolutions of these areas. Beyond facilitating data interpretation, the archival of fission track and (U-Th)/He (meta-)data in relational schemas unlocks future potential for greater integration of thermochronology and numerical geoscience techniques. The power of formatting data to interface with external tools is demonstrated through the integration of *GPlates Web Service* with *AusGeochem*, enabling thermochronology data to be readily viewed in their paleogeographic context through deep time from within the platform.

## Introduction

Low-temperature thermochronology encompasses a group of temperature-sensitive radiometric dating techniques which provide unique insights into the thermal history of Earth’s upper crust. These observations, in turn, allow scientists to constrain the timing and rate of a breadth of geological processes which can affect the thermal state of the crust over geological time, including the advection of mass and heat due to the growth of mountain belts, extensional basin formation, and long-term denudation^1–4^. Consequently, low-temperature thermochronology is an important tool for studying surface weathering processes^5^, paleoclimate^6–8^, and climate change^9,10^, as well as for constraining the formation and preservation of various natural resources, such as hydrocarbons^11,12^, hydrothermal and supergene ore deposits^13–16^, and geothermal energy fields^17,18^. In certain instances, such analyses can even record thermal events related to localised conductive heat transfer related to igneous activity^19^, volcanic eruptions^20,21^, groundwater advection^22,23^, hydrothermal fluid flow^24^, faulting and shear heating^25,26^, wildfires^27^, or meteorite formation^28^. These insights into the thermal history of the crust reflect the geodynamic, tectonic, magmatic and surficial processes which govern the evolution of our planet’s asthenosphere, lithosphere, biosphere and atmosphere.

The most commonly used low-temperature thermochronometers are the fission-track and (U-Th)/He methodologies. Like all absolute radiometric dating techniques, these systems are based on the radioactive decay of certain unstable isotope(s) to their decay product(s) at a known rate over geological time. The fission-track system, for example, is based on the formation and accumulation of microscopic damage trails (called fission tracks) in mineral grain crystal lattices due to the spontaneous fission of ^238^U atoms^29^, and the subsequent repair of these tracks via thermal annealing^30^. The (U-Th)/He system is based on the production of alpha particles (^4^He) in mineral grains during the decay chains of ^238^U, ^235^U, and ^232^Th, and the loss of ^4^He by thermally activated volume diffusion^31^. However, unlike some higher-temperature geochronology systems, such as the U–Pb or Lu–Hf systems, whose systems can be considered closed at temperatures near or above those at which the analysed geological material has crystallised or lithified^32^, the decay products (i.e., fission tracks and ^4^He) of low-temperature thermochronometers remain open at relatively low temperatures at which crustal and near-surface geological processes occur (from ~ 300 °C down to ambient temperatures depending on the mineral in question, e.g., apatite, zircon, titanite, monazite^31,33,34^). The temperature sensitivities of the fission-track and (U-Th)/He methods are further complicated by their dependence on other variables, such as cooling rate, mineral chemistry, crystal size, and radiation damage accumulation^35–38^. As a result, apparent ages (dates) produced by thermochronometers may not correspond to distinct geological events. Rather, these thermochronological data often require integration with additional measurements of kinetic parameters^35,39–42^ and numerical modelling^43^, often using thermal history modelling software^44,45^, to unravel and quantify the thermal histories that they record.

Consequently, interpreting the geological significance of thermal histories recorded by complex low-temperature thermochronology data requires placing those results in their three-dimensional geospatial, geological, and geographic context through time. These data must, therefore, be interrogated in the context of previously acquired analyses, other related geochemistry data, local geology, and modern topography. This work generally involves laborious data mining from publications and disparate repositories into private data models, bespoke to an individual analyst or research group. In the current low-temperature thermochronology data ecosystem, all of these laborious data management and synthesis tasks must then be repeated for each new study region and by each subsequent geoscientist wishing to work in that particular area. While the number of samples with low-temperature thermochronology analyses for any given region may be modest, perhaps on the order of a few thousand per continent, the variety and volume of detailed (meta-)data attributes associated with each of these results (e.g., typically > 3000 for a single apatite fission track age comprising 30 single grains and 100 confined track lengths – see Supplementary Information) and the increasing rate at which these analyses are being produced make these traditional data processing workflows ineffectual. Even in the rare instances that such low-temperature thermochronology data syntheses are published in scientific journals or data repositories, they often remain unintelligible to non-specialists due to the inherent complexities of these methodologies. Thus, new intuitive tools are needed to enable the wider geoscience community to interrogate and understand these powerful datasets.

Here, we present a novel tool for efficient geospatial examination and dissemination of global fission-track and (U-Th)/He Big Data. This robust relational low-temperature thermochronology database is an extension of the open *AusGeochem* geochemistry data platform, https://ausgeochem.auscope.org.au^46^, which enables users to upload, disseminate, interrogate, and publicise geosample metadata and secondary ion mass spectrometry U-Pb data in a geospatial context. The structured archival of detailed low-temperature thermochronology analyses in relational schemas, including fine-grained data on the individual crystal-, spot-, and track-scale (Supplementary Materials), facilitates rapid derivation of inter-data relationships, permitting data compilation, analysis, and visualisation of thousands of analyses generated by laboratories across the globe in real-time. As such, this *AusGeochem* extension presents the low-temperature thermochronology community with an unprecedented instrument for FAIR (Findable, Accessible, Interoperable, Reusable*, 45*, *46*) fission-track and (U-Th)/He data management and Big Data investigation.

## Platform functionalities

**Figure 1:**
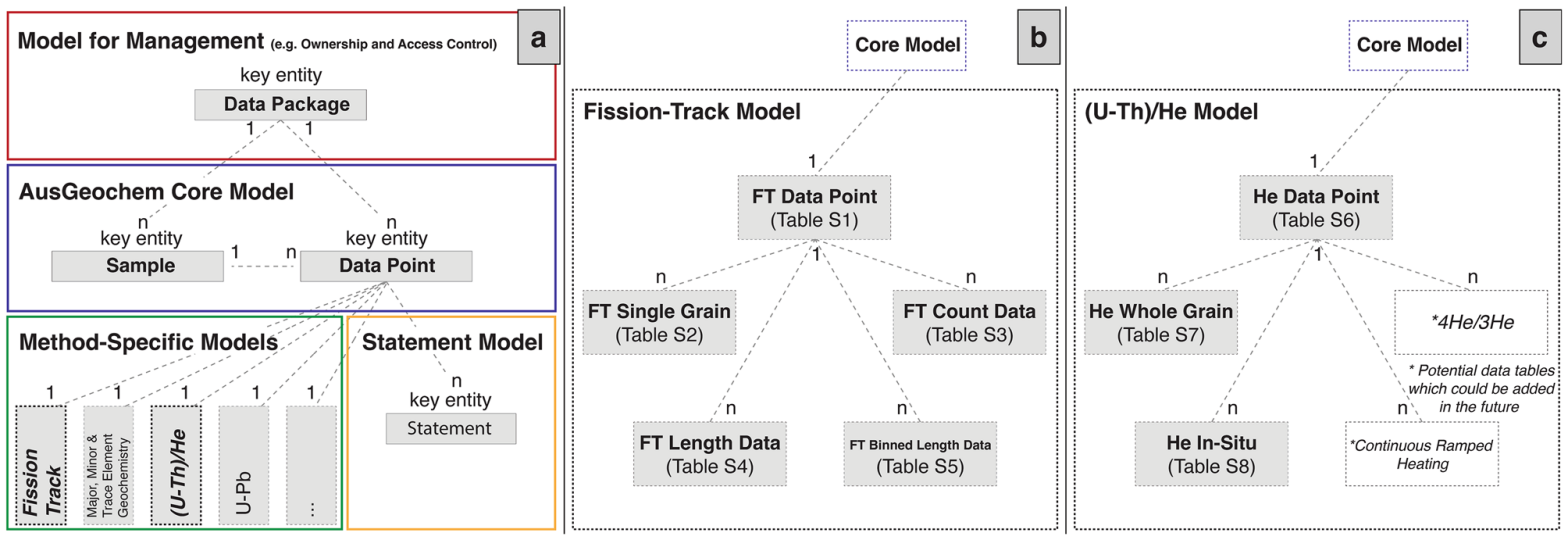
Database architecture overview of AusGeochem (**a**), the fission-track data model (**b**) and the (U-Th)/He data model (**c**). Each object within the fission-track and (U-Th)/He model represents a data table, the details of which are presented in the Supplementary Information. The fission-track data model includes a table for binned length data to accommodate legacy data in cases where detailed individual confined fission-track length data were not reported.

The relational low-temperature thermochronology database architecture of *AusGeochem* (Fig. 1) enables users to geospatially interrogate data in ways not possible using existing data portals and repositories. While existing portal and repository data model architectures constrain the user’s interaction with data to simply viewing and extracting^49^, the structured and standardised way in which fission-track and (U-Th)/He analyses are stored in *AusGeochem* enables live cross-data analytics to be performed within the platform^46^. The persistent data structure also allows for potential future developments for performing real-time computing. By making calls to the relational database, computer programs can readily retrieve the necessary data elements needed to perform a range of routines, such as the recalculation of ages based on updated decay constants, bulk thermal history modelling of regional data sets, and placing data in their palinspastic context.

**Figure 2:**
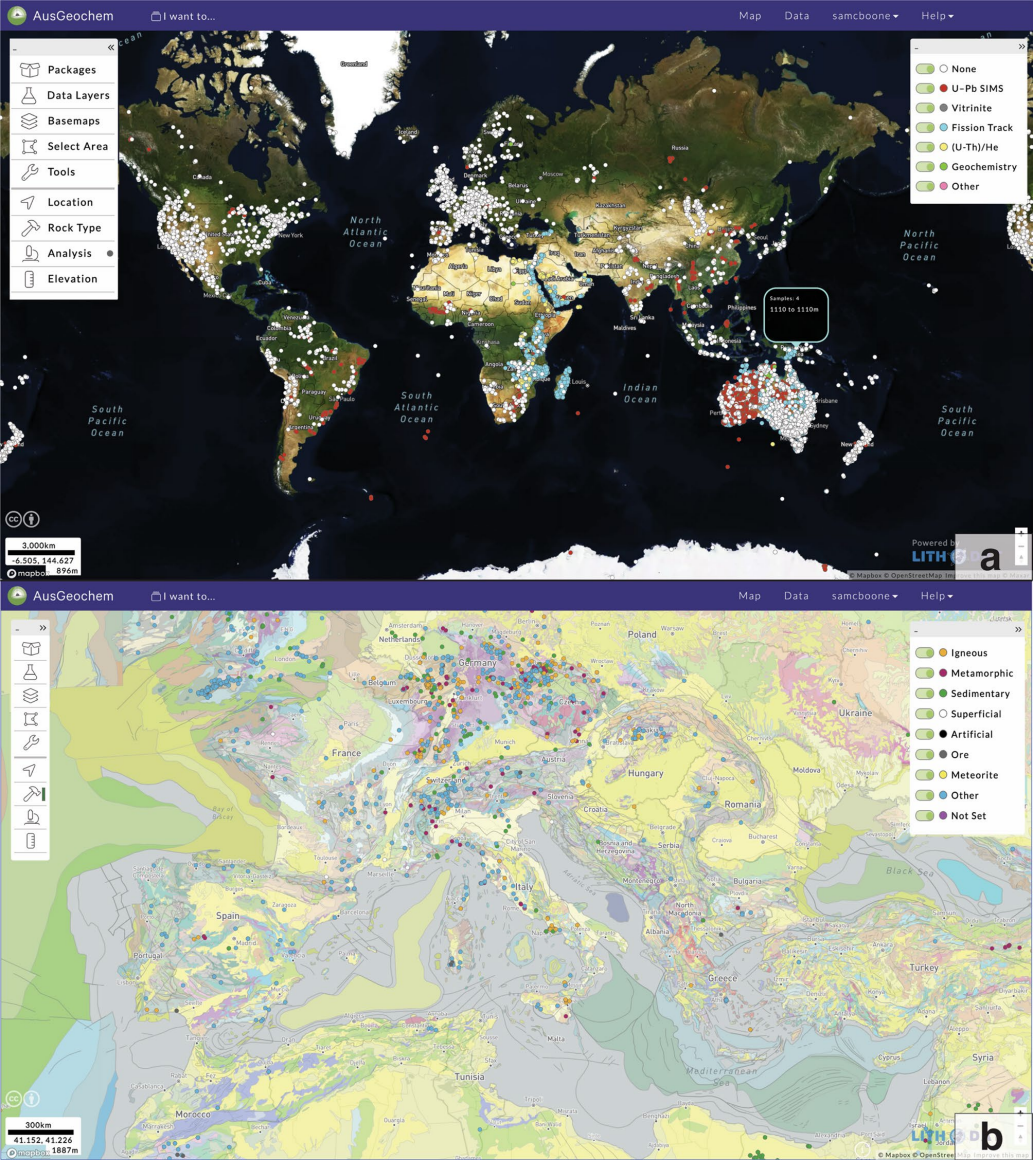
AusGeochem user interface. (**a**) Global Map View interface; (**b**) European samples coloured by rock type on geological base map. Displayed data (selected by Data Package), data types, base maps, a choice of data search tools, and a series of filters (location type, rock type, analytical method, elevation) can be selected from the toolbar on the left-hand side. Samples shown include specimens from the Museums Victoria rock and mineral collections, McNaughton sample and SIMS U–Pb data compilation, and Jones et al.^50^. Screenshots of AusGeochem (Version 2.20.79).

*AusGeochem* provides users with two interfaces to interact with data: My Data, where data can be managed, and Map View, where samples and analyses can be explored geospatially (Fig. 2). Video tutorials on how to use *AusGeochem* can be found in the User Guide section of the platform’s Help tab, or here: https://www.auscope.org.au/ausgeochem-help.

### Grassroots data ingestion

*AusGeochem* is designed as a community tool for grassroots FAIR data aggregation and dissemination, enabling data producers from around the globe to upload their own detailed analyses into the platform and make their geosample, geochemistry, geochronology and thermochronology (meta-)data publicly available. If users wish to upload compilations that include data produced by other authors, which they are welcome and encouraged to do, the original data producers must be appropriately cited by linking the relevant people, papers and reports to those data points via the Data section of the platform.

### Current state of the thermochronology database

While the aim is for the database to grow organically through the collective upload of data by platform users, a few regional datasets have been cleaned and uploaded by the authors to provide a foundation on which the database can grow. At the time of submission, > 2000 detailed apatite and zircon (U-Th)/He and apatite, zircon and titanite fission-track data from across Australia, New Zealand, Central Asia are publicly available. However, the volume of open-access thermochronology data available in the platform is expected to grow significantly over the coming year.

### Data management

Fission-track and (U-Th)/He data management is performed in the Data section of *AusGeochem*^46^, where users can upload and edit analyses individually or in bulk via a drag-and-drop tool using .csv or .xlsx data templates downloadable from within the platform. For efficient data uploading, single-grain fission-track count data and length measurements obtained using the digital fission-track analysis software *FastTracks*^51^ can be rapidly uploaded into *AusGeochem* using the ‘AusGeochem Count Data’ and ‘AusGeochem Length Data’ export formats available in *FastTracks* (version 3.3.5 and above), which can be dropped directly into the bulk upload boxes on the respective My Data tabs.

**Figure 3:**
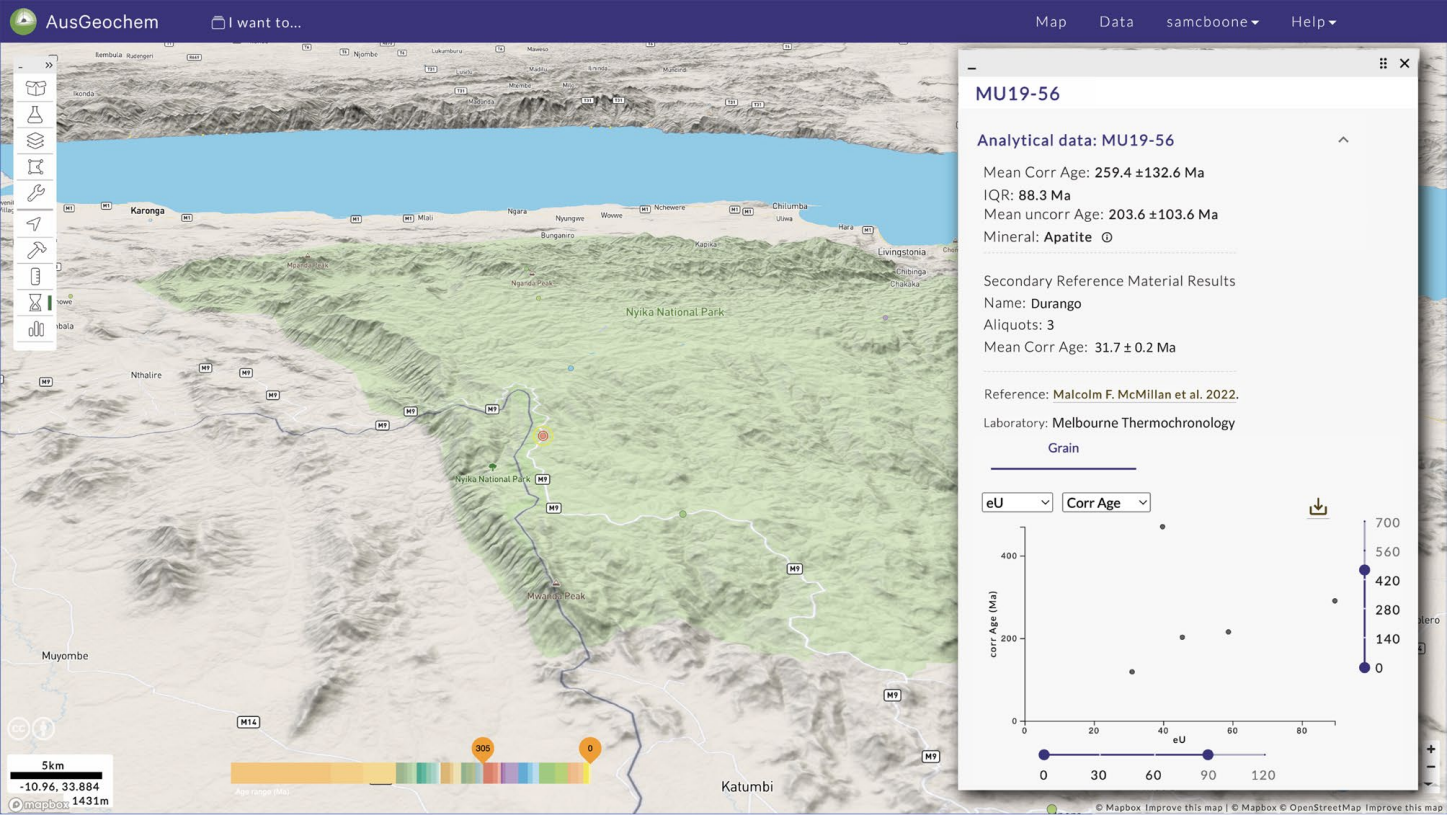
Sample metadata, analytical results, and associated secondary reference material results in the AusGeochem Map View. Unknown and secondary reference material results are related to one another via their Batch ID, a unique identifier corresponding to results obtained as part of the same set of data acquisition, allowing for rapid data quality assessment. Apatite (U-Th)/He data from McMillan et al.^52^. Screenshot of AusGeochem (Version 2.20.79).

In addition to uploading analyses of unknowns, AusGeochem users are also highly encouraged to archive associated secondary reference material results, such as age determinations of well-characterised standards in the case of (U-Th)/He data. This is critical as it allows platform users to independently assess data quality based on the reproducibility of well-characterised reference materials of known composition and/or age. For fission-track analysis, this includes reporting analyst-specific zeta-calibration factors, neutron irradiation parameters and dosimeter fission track densities in the case of External Detector Method-generated data, or fission-track age or trace element concentration determinations for reference materials in the case of absolute fission-track dating via LA-ICP-MS or EPMA. Once uploaded, the associated reference material results for a given unknown analysis can then be quickly viewed under the Analytical Data pop-up when that sample is selected in Map View (Fig. 3).

### FAIR data via IGSN and DOI minting

Leveraging Lithodat’s DataCite membership, *AusGeochem* enables users to mint persistent identifiers, such as International Generic Sample Numbers (ISGNs)^53^ and Digital Object Identifiers (DOIs), to ensure their samples and analyses are more FAIR (Findable, Accessible, Interoperable, Reusable). Once finalised and made public, data packages can thus be made citable by minting a DOI. The DOI can then be included in associated publications and reports, providing a direct link to data stored in *AusGeochem* where they can be interrogated and extracted.

### Data interrogation in map view

*AusGeochem’s* Map View user interface enables geological sample and geochemistry data to be explored in their geospatial context. Users can select data types and perform further data filtering in Map View using a combination of lithological, mineralogical, elevation, and method-specific attributes (Fig. 2b).

**Figure 4:**
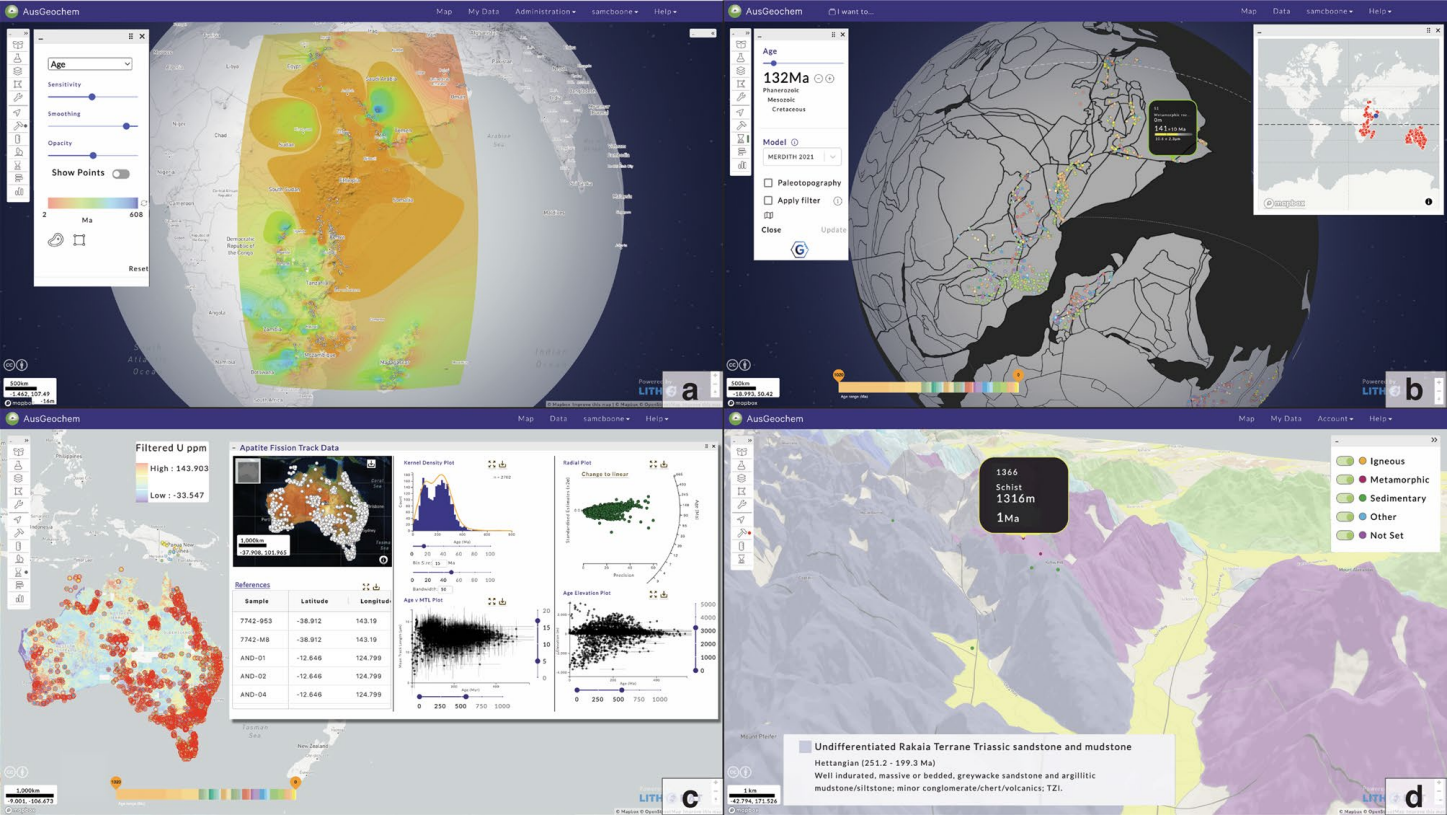
A range of base maps and data projections in AusGeochem. (**a**) Data interpolation tool, contouring apatite fission-track ages in eastern Africa in spherical projection. (**b**) Apatite fission-track from central Gondwana in their 132 Ma paleogeographic context according to the plate tectonic model of Merdith et al.^54^. (**c**) Apatite fission-track dashboard synthesising data from across Australia^55^ viewed on a radiometrics (U) layer. Dashboards enable multiple data points, chosen using the Select Area tool, to be collectively interrogated via a range of interactive maps, plots and tables. The inset map and all plots can be downloaded as editable svg files. A succinct data table can also be downloaded as a csv file. A downloadable reference list for all selected data points is also automatically generated, ensuring data sources can be appropriately cited. (**d**) Apatite (U-Th)/He data by rock type on geological map viewed in a 3D perspective. Geological information can be viewed by clicking on a unit of choice. All figure elements were exported from AusGeochem (Version 2.20.79) before being combined and annotated in a third-party graphics editor program.

A selection of base maps and map projections are available, including satellite images, dark and light contrast maps, topography, and global geology, all of which can be viewed in a Web Mercator (EPSG:900913) or spherical projection (Fig. 4). Where available, other regional base maps, such as regional gravity, magnetic, and heat-producing radioactive elemental concentration data layers can also be viewed (Fig. 4c). Data can also be viewed in their 3D topographical context. In this way, any base map layer can be draped over the vertically exaggerated digital elevation model (e.g., geology, Fig. 4d).

**Figure 5:**
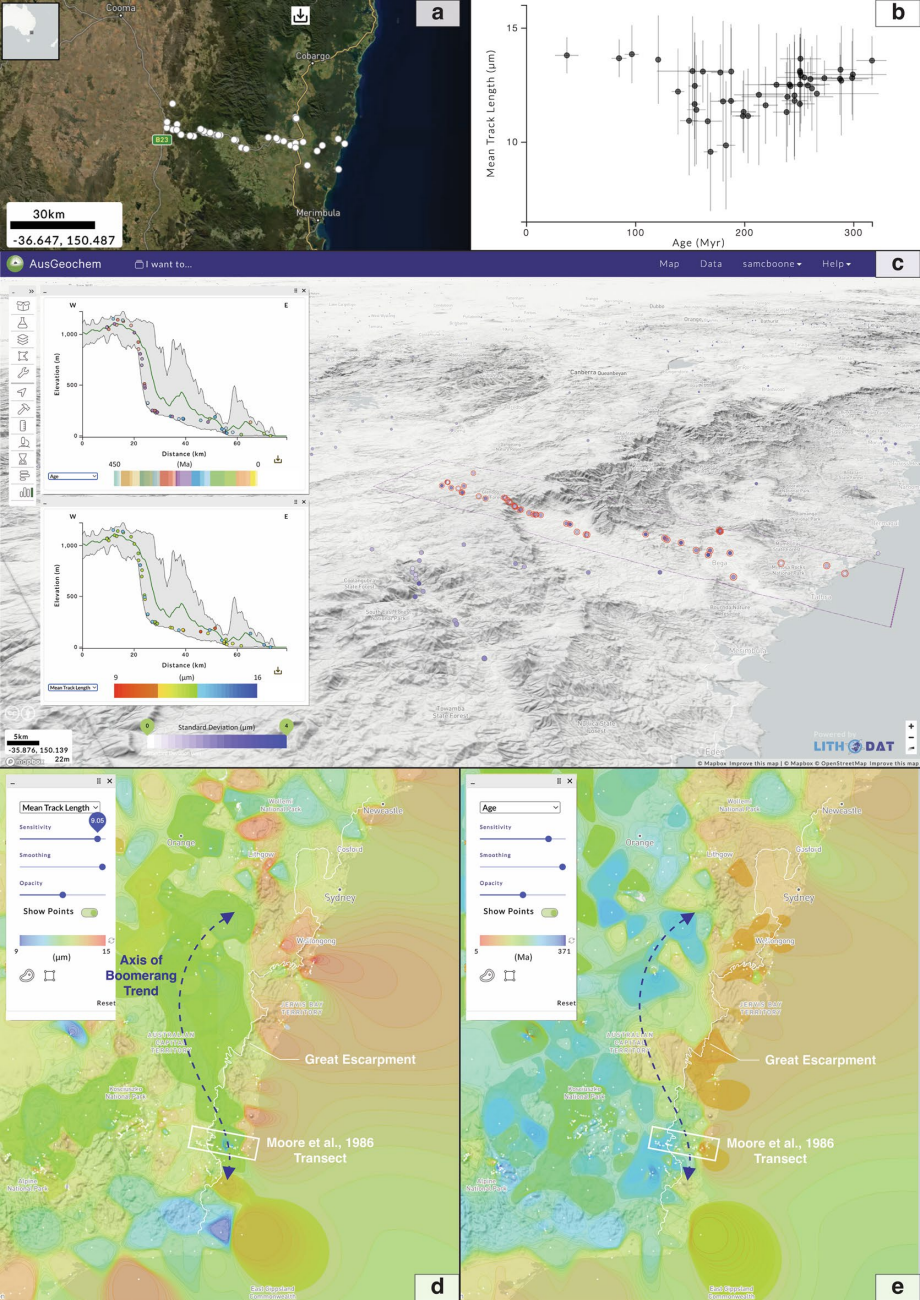
Apatite fission-track data trends along the eastern Australian margin. (**a**) Sample locations and (**b**) age versus mean track length (Boomerang) plot of fission-track transect of Moore et al.^60^ and McMillan et al.^72^. (**c**) Swath profiles of combined apatite fission-track data transect of Moore et al.^60^ and McMillan et al.^72^ showing relationship between age, mean track length, and standard deviation. Regional interpolation of mean track lengths (**d**) and apatite fission-track ages (**e**) from southeast Australia^55,60,72^ show how the fortuitous relationship between apatite fission-track data and modern topographic expression of the rifted Great Escarpment breaks down north of the classic transect of Moore et al.^60^. All figure elements were exported from AusGeochem (Version 2.20.79) before being combined and annotated in a third-party graphics editor program.

Simple sample and analytical information can be retrieved via a pop-up window by hovering the cursor over a sample point on the map (Fig. 2b), while more detailed (meta-)data can be obtained by selecting a given sample (Fig. 3). A range of data interrogation tools are also available. These include a data interpolation tool, which generates 2D contoured heat maps for selected variables like age or mean track length (Fig. 4a) and a swath profile tool for investigating the relationship between selected attributes and topography (Fig. 5c). Using the Multi-Select tool, numerous samples on the map can be selected simultaneously allowing for real-time data synthesis and visualisation. By dragging a polygon over an area of interest, a simplified table summarising the selected data points can be queried. Here, users can then select from a range of method-specific dashboards (e.g., fission-track dashboard, Fig. 4c) which synthesise the selected data in real-time via comparative plots and derivative maps relevant for each methodology.

### Fission-track and (U-Th)/He dashboards

The bespoke fission-track and (U-Th)/He dashboard provides users with intuitive tools to interrogate select collations of thermochronology data via a range of maps, tables, and interactive plots (Fig. 4c), all of which can be exported in publication ready formats. Dashboard plots include an age histogram, radial plot, age versus mean track length scatterplot (boomerang plot), age versus elevation plot, and flexible scatterplots that allows users to plot fission-track ages versus geochemical parameters, all of which can be downloaded as .svg files for further editing if needed. To ensure that data producers are appropriately credited, the dashboards also provide a downloadable reference list associated with the selected data (Fig. 4c).

### Application programming interface (API)

*AusGeochem* is equipped with an Open REST Application Programming Interface (API), allowing any developer to build clients that can interact with the platform to, for example, automatically upload or retrieve data from its database, add enhanced data visualisation tools and create direct links to analytical equipment. Potential uses of this powerful tool for the low-temperature thermochronology community could include the development of clients enabling automated data upload using common data formats (e.g., TrackKey^44^, HeFTy^45^, QTQt^54^) and automated data retrieval for bulk thermal history modelling or landscape evolution modelling (e.g., PECUBE^57^).

API documentation and user instructions on how to access the API can be found under the Help tab in *AusGeochem*.

### GPlates web service integration

The power of structuring data to interface with external tools through the platform API is demonstrated through the integration of the *GPlates Web Service* with *AusGeochem*. The *GPlates Web Service* is developed by the EarthByte Group at the University of Sydney as a part of the *GPlates*^58^ project, which allows users to access *GPlates*’ plate tectonic reconstruction functionality via HTTP requests over the internet or local networks. The *GPlates Web Service* places geological samples and their associated geochemistry data, retrieved via the *AusGeochem* API, in their paleogeographic position according to published plate tectonic reconstruction models (Fig. 4b). Users can then inspect and analyse data over a range of paleo-rasters, such as paleotopography^59^.

### Data extraction and crediting data sources

Selected data can be extracted from both Map View and My Data and exported in .csv and .xlsx formats, with a shapefile exporting functionality under development. Users are also encouraged to code their own routines for streamlined fission-track and (U-Th)/He data upload and download directly to and from third-party software, repositories, or databases using *AusGeochem’s* open REST API (see below).

A critical component of the transition to a more FAIR thermochronology data ecosystem is ensuring that both data producers and compilers receive due credit through appropriate citations, in line with FAIR data principles^47^. Assuming users properly link their data to their associated references upon upload, reference information for each data point in AusGeochem can then be found in their metadata under “Literature” in the Data Section, or in the pop-up window when an individual sample is selected on the Map (Fig. 3). When multiple data are selected in Map View using the multi-select tool, a reference list of all data sources is automatically generated which can be downloaded from the dashboard view (Fig. 4c).

## Geospatial interpretation of thermochronology data

The relational fission-track and (U-Th)/He data models of *AusGeochem* provide novel tools for dynamic geospatial interrogation of low-temperature thermochronology data. To illustrate this, three regional case studies from around the globe are briefly presented below. Through these examples, the importance of interrogating large compilations of fission-track and (U-Th)/He data in their geospatial context is demonstrated using the built-in tools currently available within *AusGeochem*. While these examples highlight regional fission-track and (U-Th)/He methods applications in intracontinental rifting, continental breakup, and passive margin settings, it is stressed that *AusGeochem* is designed to geospatially interrogate low-temperature thermochronology data at all scales, across the full breadth of geological environments. The case studies are followed by a discussion of potential future developments and powerful applications of this relational thermochronology data platform.

Readers can interrogate and utilise the detailed regional fission-track and (U-Th)/He datasets discussed below, which are freely available on *AusGeochem*. These compilations include data produced by the authors, as well as a significant number of detailed data mined from the literature. These compilations are meant to serve as a foundation to the thermochronology database, with the intention and hope that future users will continue to add detailed fission-track and (U-Th)/He datasets, both legacy and new.

### Thermochronological insights into australian passive margin evolution

Since Moore et al.^60^ first reported an apparent relationship between the modern topography of the southeast Australian margin and apatite fission-track data observed along a transect orthogonal to the coast, low-temperature thermochronology has routinely been used to constrain the denudation history of rifted continental margins^61–63^, leading to the development of a range of tectonic models for passive margin development^64,65^. Specifically, Moore and colleagues^60^ observed a transition from relatively young apatite fission-track ages and long confined track lengths at the coast, through to moderate ages with short track lengths on the erosional escarpment, and eventually to old ages and long track lengths atop the escarpment, which they collectively attributed to a Late Cretaceous rift-related thermal event that preferentially reset apatite fission-track data along the southeast Australian margin (Fig. 5a, b). This concave-up “boomerang trend” observed in the apatite fission-track age versus mean track length data (Fig. 5a) has since become a diagnostic signature of progressive thermal overprinting of an older background thermal history by a younger cooling event^66^, and frequently applied to interpreting the thermochronology of rifted continental margins around the world^65,67–69^.

However, in light of more regional-scale thermochronology data syntheses and integration with other geological evidence, the underlying assumption that the development of modern topography implicitly relates to the underlying apatite fission-track data has recently been called into question in some instances^70–72^. Even along the southeast Australian margin where it was first observed, the relationship between fission-track data and modern rift margin topography breaks down when viewed in its regional context^72^. Instead, north of the original apatite fission-track data transect of Moore et al.^60^ which fortuitously corresponds with the modern topographic profile of the Australian margin, the “boomerang trend” diverges inland (Fig. 5c, d). This requires that the apparent pattern in thermochronology data predates the topographic development of the present Australian margin^72^, bringing into question the assumed genetic link between low-temperature thermochronology data and the modern topographic expression of rifted continental margins worldwide. While not claiming that this re-interpretation may necessarily apply to passive rifted margins elsewhere, it does act as a cautionary tale, encouraging the scrutiny of low-temperature thermochronology datasets from other passive margins in their regional context, which can be readily enabled using the *AusGeochem* platform.

### Thermochronological imaging of tectonic inheritance in Kenya

**Figure 6:**
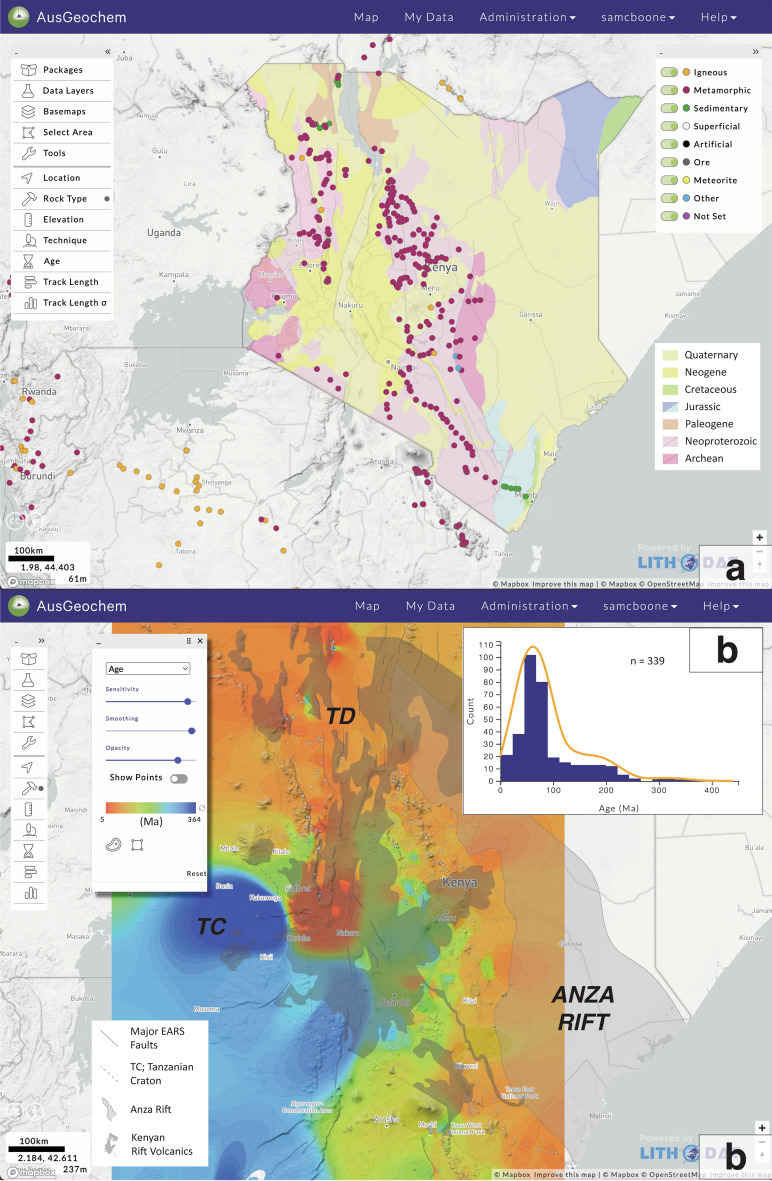
Simplified geology of Kenya (**a**), with interpolation (**b**) and distribution (**c**) of apatite fission-track ages. Mean track length interpolation and data sources are listed in the Supplementary Material (Fig. S1 & Table S9). All figure elements were exported from AusGeochem before being combined and annotated in a third-party graphics editor program. Distribution of Kenyan rift volcanic rocks from Beicip^80^. TC = Tanzanian Craton; TD = Turkana Depression; EARS = East African Rift System. All figure elements were exported from AusGeochem (Version 2.20.79) before being combined and annotated in a third-party graphics editor program.

The East African Rift System (EARS) has long attracted geoscientists looking to employ low-temperature thermochronology to investigate intracontinental rift processes. This has resulted in a dense coverage of low-temperature thermochronology analyses, particularly apatite fission-track data in Kenya (Fig. 6). When viewed regionally in *AusGeochem*, these data reveal the tectonothermal signature of a long history of superimposed tectonic events imprinted into the East African crust. Taken together, these data provide an informative view into the geological processes that have most affected the cooling history of rocks now exposed at the surface, often reflecting the timing and rate of erosional denudation in response to tectonism.

The oldest apatite fission-track dates (204 ± 11 to 364 ± 18 Ma) within Kenya are found in Archean rocks of the Tanzanian Craton and Neoproterozoic mobile belts of central Kenya. While the geological significance of these dates is poorly understood, in part due to a dearth of confined track data collected using modern techniques (Fig. S1), the preservation of these old ages attest to the relative stability of the Tanzanian Craton since the Palaeozoic. The most prominent tectonothermal signature documented in the region, however, is recorded by a profusion of ~ 100–50 Ma ages (orange in Fig. 6b) observed in a linear band from southeast to northwest Kenya. These have been interpreted as reflecting > 2.5 km of rift shoulder uplift and denudation along the margins of the Anza Rift, a failed Cretaceous-early Paleogene rift system running northwest from the Kenyan coast^73^ that once may have extended across the Turkana Depression into South Sudan^74^. Surprisingly, few data yield Neogene cooling ages (red in Fig. 6b) associated with the development of the Miocene-recent Kenyan sector of the EARS, despite the rift dominating the modern geomorphology of the region. This suggests EARS-related rift-shoulder uplift has been insufficient to exhume rocks that have cooled entirely through the temperature sensitivity range of the apatite fission-track system. Only in the Turkana Depression of northern Kenya and localised along a basin-bounding normal fault in central Kenya are Neogene cooling ages recorded, with the former attributed to a combination of higher palaeogeothermal gradients and increased basin margin uplift due to lower flexural rigidity of the highly attenuated crust there^75^ and the latter associated with localised hydrothermal fluid activity^76^.

However, some of the most important insights into what governs the low-temperature thermochronology of the upper crust in intracontinental settings can be found when Kenyan apatite fission-track data are juxtaposed against the regional geology (Fig. 6). Despite the surface geology being largely composed of aerially-extensive Neogene volcanic rocks (Fig. 6b), a notable absence of regional thermal rejuvenation of apatite fission-track data by the intense magmatic history of the Kenyan Rift becomes readily apparent. Since the early Miocene, and beginning even earlier (Paleogene) in Turkana, the interplay of plume activity and lithospheric thinning has resulted in the emplacement of a perfuse volume of extrusive volcanic rocks in Kenya (924,000 km^3^ ^77^) and the addition of unknown, but no doubt significant, amounts of igneous material in the sub-surface^78,79^. Yet, apatite fission-track data yield very few Neogene ages recording the influence of conductive heating related to the extensive magmatic history of the rift. This is consistent with previous assertions that the conductive thermal effect of magmatism on low-temperature thermochronology is restricted to similar length scales of individual lava flow thicknesses and intrusive body dimensions^1,67,69^. By readily placing Kenyan thermochronology in its geological context using *AusGeochem*, the utility of regional apatite fission-track assays for constraining the spatio-temporal denudational response to upper crustal extensional strain is demonstrated, even in a magmatic intracontinental rift setting.

### Geomorphological evolution of the red sea rift escarpments

Long-term denudation rates estimated from low-temperature thermochronology can provide important constraints for the geomorphic evolution of rifted margins^65^. Such is the case for the conjugate Nubian-Arabian margins, whose transformation from being topographically subdued in the Early Oligocene to displaying their modern steep coastal escarpments is attributed to the development of the Red Sea rift system since the Late Oligocene^81^.

**Figure 7:**
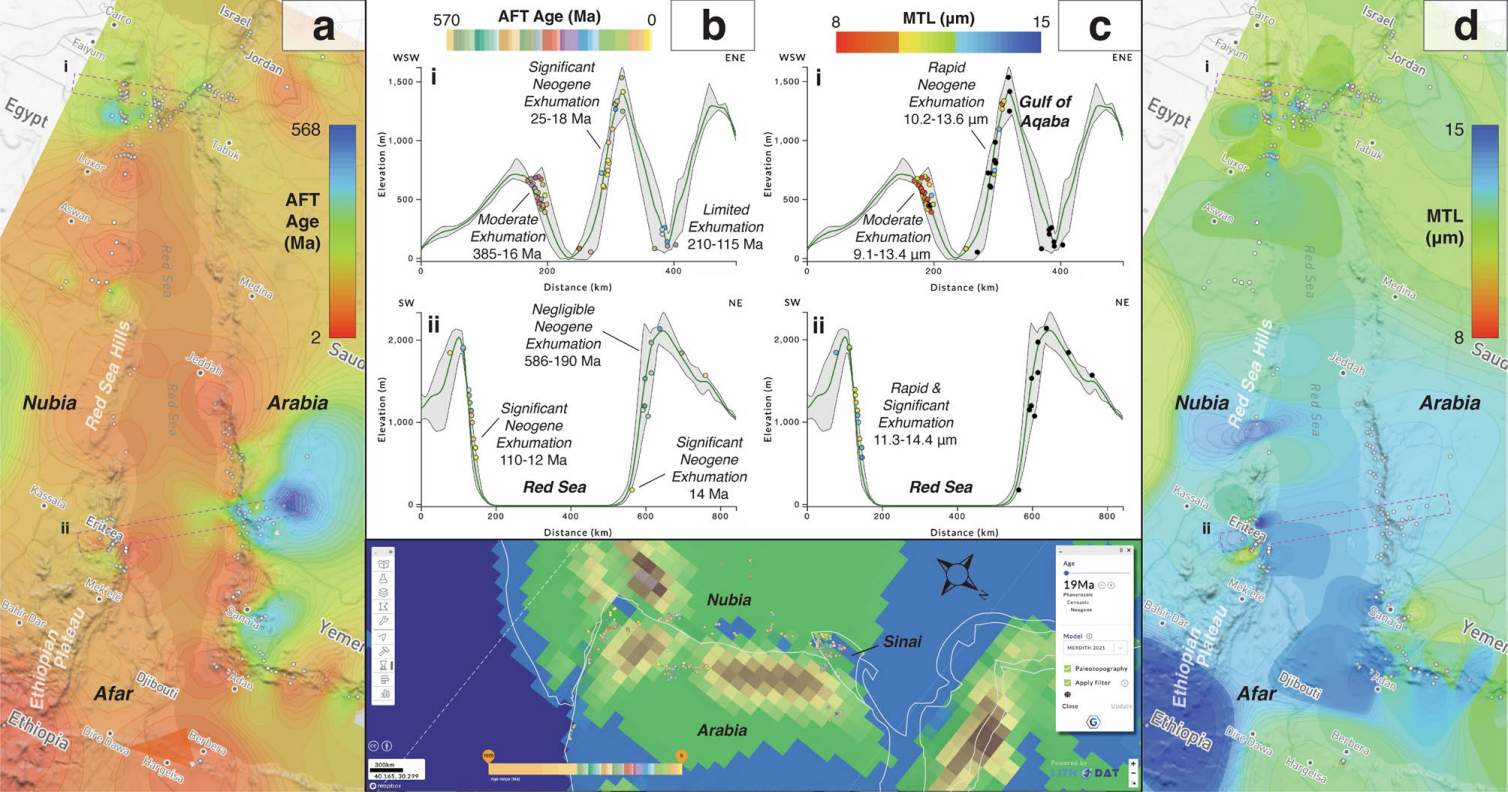
Relationship between apatite fission-track data and geomorphology along the Red Sea Margins. Data sources are listed in the Supplementary Materials (Table S10). (**a**, **b**) Fission-track age and (**c, d**) mean track length (MTL) interpolations and swath profiles were exported from AusGeochem before being annotated in a third-party graphics editor program. Black dots in swath profiles indicate no data. (**e**) Red Sea apatite fission-track ages in their 19 Ma paleogeographic position, according to the plate tectonic model of Merdith et al.^54^, and viewed against the paleotopography model of Cao et al.^59^. Plate boundaries shown in light grey. Dark blue = deep marine; light blue = shallow marine; green = land; tans-to-browns = moderate-to-high topography. All figure elements were exported from AusGeochem (Version 2.20.79) before being combined and annotated in a third-party graphics editor program.

**Figure 8:**
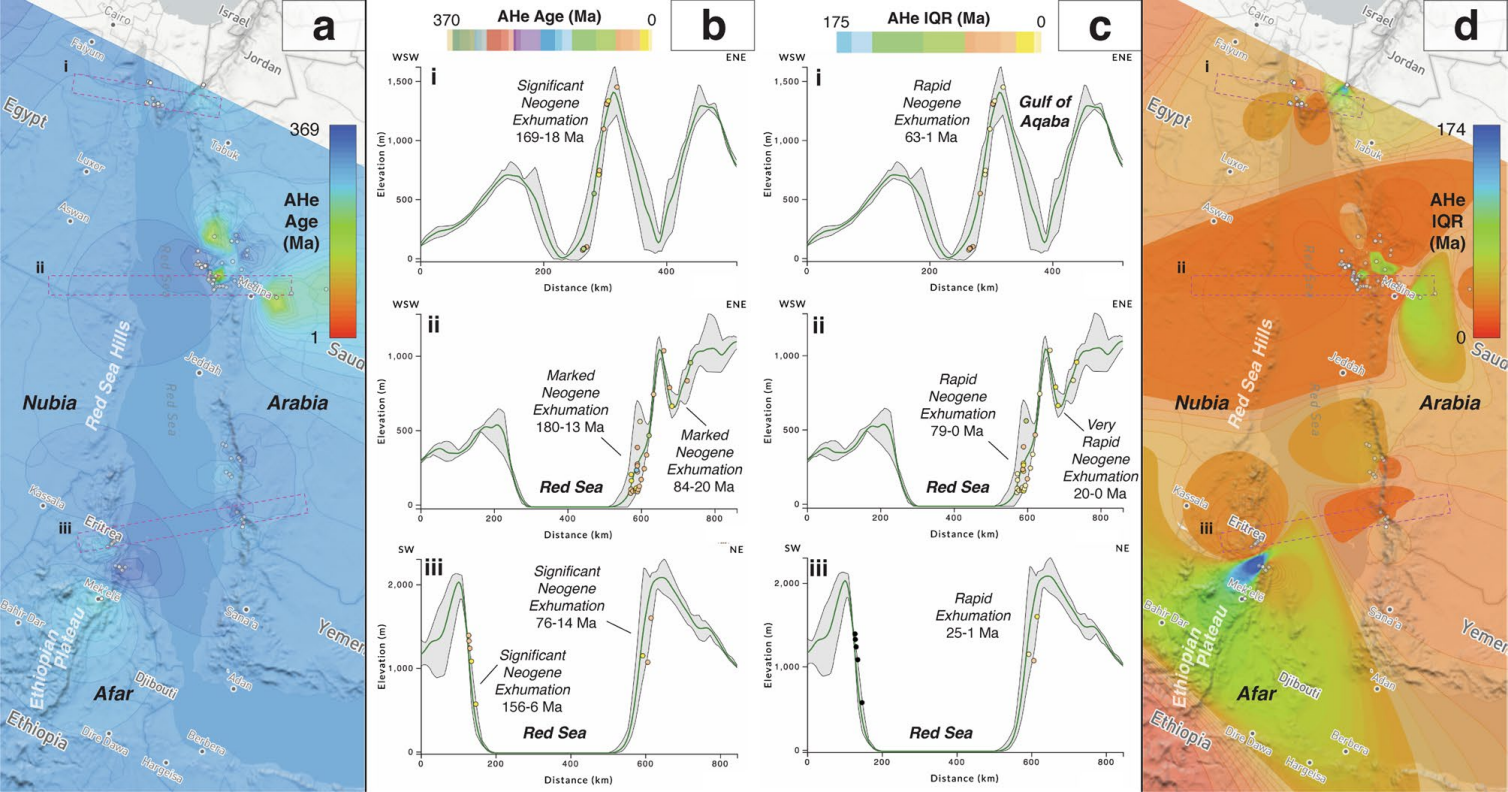
Relationship between apatite (U-Th)/He data and geomorphology along the Red Sea Margins. Data sources are listed in the Supplementary Materials (Table S10). (**a**, **b**) Mean apatite (U-Th)/He age and (**c**, **d**) interquartile range (IQR) interpolations and swath profiles were exported from AusGeochem before being annotated in a third-party graphics editor program. Black dots in swath profiles indicate no data. All figure elements were exported from AusGeochem (Version 2.20.79) before being combined and annotated in a third-party graphics editor program.

Despite an uneven distribution of low-temperature thermochronology data along the Red Sea, marked differences in apatite fission-track (Fig. 7) and (U-Th)/He (Fig. 8) data trends from the Nubian and Arabian margins reflect their disparate geomorphologies^82^, as clearly shown when viewed in *AusGeochem*. The Nubian margin, whose low-lying deserts are separated from the coast by the narrow Red Sea Hills (500 m mean elevation), contrasts starkly with the broad highlands (1,000–1,500 m mean elevation) of the Arabian margin that attains heights of up to 3,200 m at its southern end. This physiographic asymmetry is reflected in the low-temperature thermochronology data. Whereas the Arabian margin yields Oligo-Miocene apatite fission-track and (U-Th)/He ages and long mean track lengths along much of its rift escarpment, indicative of rapid exhumation during that time period, the Nubian margin exhibits significantly older thermochronological ages along most of its topographically subdued profile. The observed asymmetry in Oligo-Miocene cooling is consistent with predicted differences in the paleotopographic expression of the margins at that time (Fig. 7e). According to the model of Cao et al.^59^, samples from the proto-Nubian margin were located in a low-lying, subsiding region, in places inundated the early Miocene, in constrast to the highlands of the incipient Arabian margin (Fig. 7e). Only at the southern extent of the Nubian margin along the base of the > 2,000 m Ethiopian Plateau is pronounced Miocene cooling observed, where its tectonothermal evolution related to the opening of the southern Red Sea is compounded by additional plume driven magmatism, hydrothermal fluid flow and incipient lithospheric rupture in the Afar^83,84^. Debate continues as to why the conjugate margins of the Red Sea rift evolved so differently^85^, with explanations ranging from northeast tilting of Arabia due to its collision with Eurasia^86^ to dynamic topography gradients^87^. Nevertheless, the pronounced differences in the timing and rate of rift margin exhumation quantified by low-temperature thermochronology provide valuable observations with which to test these geodynamic hypotheses.

## Future outlook

The advent of dynamic relational fission-track and (U-Th)/He databases heralds the beginning of a new era of structured Big Data in the field of low-temperature thermochronology. By methodically archiving detailed fission-track and (U-Th)/He (meta-)data in structured schemas, intractably large datasets comprising 1000 s of analyses produced by numerous laboratories from around the globe can be readily interrogated in new and powerful ways. The collective use of a single community-designed data reporting schema will also aid the low-temperature thermochronology community to converge on more consistent data reporting practices, better connecting data producers and users. The ability to geospatially search available fission-track and (U-Th)/He data and immediately access all of the associated detailed analyses would significantly streamline future data mining, while also providing a means to rapidly identify data gaps for future research.

Yet, the ability to rapidly visualise and synthesise fission-track and (U-Th)/He analyses in a geospatial context is but a hint at the true potential of archiving low-temperature thermochronology data in a structured database architecture. With detailed thermochronology data stored in relational schemas, the step to developing scripts capable of re-calculating and re-modelling analyses using user-defined constants and kinetic algorithms is relatively straightforward. Such a future advancement would enable analyses determined using different parameters and constants to be equated and compared across regional- to global scales. Similarly, *AusGeochem’s* open API could be leveraged to automate data extraction to interface with other existing numerical geoscience tools, such as thermal history or numerical landscape evolution modelling codes and applications. The power of the API is demonstrated through the integration of *GPlates Web Service* with *AusGeochem*, enabling fission-track and (U-Th)/He analyses to be readily interrogated in plate tectonic and paleotopographic frameworks through deep time in a way never before possible. An additional platform extension currently under development which will allow thermal history modelling results to be stored in *AusGeochem* alongside raw data will only further expand the utility of this dynamic thermochronology data tool.

However, to take full advantage of the wealth of invaluable thermochronology data and maximise their use for numerical modelling techniques, *all* global thermochronology data, old and new, must be made discoverable and publicly available in structured, machine-readable formats via relational platforms like *AusGeochem*. This will inevitably involve the significant task of aggregating the plethora of global legacy thermochronology data. Fortunately, the volume of legacy data is finite, and data compilation need only be performed once if done thoroughly and made publicly available. The advent of “web scraping” tools^88^ could also help to automate legacy data mining already stored in online repositories, though these still require a degree of manual cleaning. It is equally important that prospective data are made open-access and reported in full detail following community-guided best practices^31^. While *AusGeochem* provides tools for more efficient data ingestion through its drag-n-drop uploader and API, an unavoidable degree of manual data wrangling will nevertheless be required for the foreseeable future. Data producers and compilers must therefore be incentivised to proactively contribute their datasets to open access platforms, like *AusGeochem*, through proper data source attribution. Thus, a concerted data-culture transformation must take place in our community if we wish to truly usher in a new era of thermochronology Big Data.

Readers may freely register and explore *AusGeochem* at https://ausgeochem.auscope.org.au.

## Methods

### AusGeochem geochemistry data platform

In 2019, a consortium of Australian university research laboratories named the AuScope Geochemistry Network set out to build a collaborative platform for the express purpose of collating, preserving, and disseminating geochemistry and geochronology data. In partnership with geoscience-data-solutions company Lithodat Pty Ltd, the open, cloud-based *AusGeochem* platform (https://ausgeochem.auscope.org.au^46^) was developed to simultaneously serve as a geosample registry, a geochemical data repository, and a data interrogation tool. In collaboration with method-specific advisory groups of geochemistry experts and adopting established international data reporting practices, community-agreed upon data schemas have been developed for rock and mineral geosample metadata, secondary ion mass spectrometry U–Pb analysis, fission-track and (U-Th)/He analysis. These will be accompanied progressively by additional data models for laser ablation inductively coupled mass spectrometry (LA-ICP-MS) U–Pb and Lu–Hf, oxide and major, minor and trace element geochemistry, and Ar–Ar, all currently under development, with the intention of additional method-specific geochemistry data models to follow.

### Fission-track and (U-Th)/He database scope and development

Schemas for the *AusGeochem* fission-track data model (Tables S1-S5) and (U-Th)/He data model (Tables S6-S8) were designed with the aim of accommodating retrospective and prospective datasets alike. Such flexibility requires that the data schema can archive analyses produced across mineral systems and via the breadth of historical fission-track and (U-Th)/He techniques. For the fission-track system, this includes results generated from the outdated population method^89^, conventional external detector method (EDM, e.g., *32*), and state-of-the-art in-situ fission track analysis using laser ablation inductively coupled plasma mass spectrometry (LA-ICP-MS, e.g., *89*, *90*) or electron probe microanalysis (EPMA), in the case of analysis of relatively U-rich mineral assemblages like zircon^92^. For the (U-Th)/He system, the current version of the (U-Th)/He relational data model is built to accommodate whole-grain and in-situ age determinations. While the current *AusGeochem* (U-Th)/He relational data model cannot accommodate results generated via the emerging ^4^He/^3^He or Continuous Ramped Heating methods^31^, its flexible architecture would allow for straightforward integration of bespoke data tables for these analysis types, should they be designed in the future (Fig. 1).

The relational FT database was also designed to handle wildly varying degrees of (meta-)data reporting granularity found across the gamut of published thermochronology studies. So, in addition to archiving whole-rock fission-track results (Table S1), the model can accommodate both detailed single-grain fission-track count (Table S3) and age data (Table S2), when available. Similarly, users are able to upload both comprehensive confined fission-track length data (Table S4) generated using digital microscopy software (e.g., *FastTracks*, *48*), or legacy binned confined track data reported simply as length histograms without the corresponding detailed length parameters (Table S5). While the fission-track model is strictly designed to accommodate fission-track analyses sensu stricto, associated in-situ geochemical analyses obtained via EPMA or LA-ICP-MS trace element analysis can be archived and related to fission-track results on a per-sample, per-grain, and per-spot basis in the linked Major, Minor and Trace Element Geochemistry Data Model (Fig. 1).

The (meta-)data fields and corresponding units of the fission-track and (U-Th)/He data tables were designed after global community-agreed reporting recommendations. The (U-Th)/He data model and corresponding tables (Tables S6-S8) were designed following the data reporting best-practices of Flowers et al.^31^. While the fission-track model (Tables S1-S5) was designed after the recommended data reporting practices currently being prepared for submission to *Geological Society of America Bulletin* special edition on “Reporting and Interpretation of Fission-Track Chronology Data”, with which co-authors of this article (B.K, S.B., A.G., and M.D.) are involved.

### AusGeochem data platform architecture

The fission-track and (U-Th)/He data models presented herein were designed as an extension to the *AusGeochem*^46^ relational database architecture (Fig. 1). The *AusGeochem* platform comprises four integral components. At the highest level is the Model for Management, which enables users to manage data privacy control on a per dataset basis, called ‘Data Packages’ in *AusGeochem*. Upon data upload, users have the option to keep their unpublished data private, disseminate their data to select collaborators, or make their data open access. This user-defined privacy control system gives analysts the ability to upload and interrogate their unpublished data in the context of thousands of results from around the globe, whilst keeping their data private until results are ready for publication. However, uploaded data is subject to *AusGeochem’s* open data policy, which limits the data privacy embargo to two years plus the option for an automatic 1-year privacy extension.

Geological mineral and rock samples, along with their associated information, are stored in the Core Model. From here, samples can be linked to Method-Specific analyses, such as those stored in the fission-track or (U-Th)/He data models. Each sample can have multiple related method-specific Data Points, each of which represents a particular analysis performed on that geosample. In other words, an individual sample can be linked to multiple analyses, such that the reanalysis of a given sample would simply be uploaded as a new method-specific Data Point (e.g., an FT Data Point). This 1-n relationship between the Core Model and the Method-Specific Models also allows geochemical data of different kinds to be related on a per-sample or per-aliquot basis. This is particularly relevant for fission-track analyses which are often accompanied by other complementary geochemical measurements. For example, fission track results can be linked to corresponding electron probe microanalysis, LA-ICP-MS trace element, and U–Pb data stored in other Method-Specific Data Models via their shared Sample, Mount, Grain, and in some cases, Spot IDs (Fig. 1). Thus, *AusGeochem* will also be able to accommodate the archival of double- and triple-dating results involving combined fission-track, U–Pb, and (U-Th)/He determinations^91,93,94^.

The fourth component of the *AusGeochem* platform is the Statement Model, which enables advanced on-the-fly analytics to be performed across all Data Points and data types. Here, the ‘statement(s)’ derived for each Data Point, such as age, chemistry, isotopic ratio, or time–temperature history, are stored^48,56,90^.

## Supplementary Information

Supplementary Information 1.

Supplementary Information 2.

Supplementary Information 3.

## Data Availability

The *AusGeochem* data platform and the low-temperature thermochronology data extension presented here are open-access and freely available to users from around the world at https://ausgeochem.auscope.org.au. Relational fission-track and (U-Th)/He data tables, including descriptions and units of all attribute fields are available in the main text or the supplementary materials. API documentation and user instructions on how to access the open REST Application Programming Interface (API) can be found under the Help tab in *AusGeochem*.

## References

[1] Ehlers TA (2005). Crustal thermal processes and the interpretation of thermochronometer data. Rev. Mineral. Geochem..

[2] Stockli DF (2005). Application of low-temperature thermochronology to extensional tectonic settings. Rev. Mineral. Geochem..

[3] Reiners PW, Brandon MT (2006). Using thermochronology to understand orogenic erosion. Annu. Rev. Earth Planet. Sci..

[4] Schildgen TF, van der Beek PA (2019). The Application of Low-Temperature Thermochronology to the Geomorphology of Orogenic Systems.

[5] Ault AK, Gautheron C, King GE (2019). Innovations in (U–Th)/He, fission track, and trapped charge thermochronometry with applications to earthquakes, weathering, surface-mantle connections, and the growth and decay of mountains. Tectonics.

[6] Kohn BP, Pillans B, McGlone MS (1992). Zircon fission track age for middle Pleistocene Rangitawa Tephra, New Zealand: stratigraphic and paleoclimatic significance. Palaeogeogr. Palaeoclimatol. Palaeoecol..

[7] Shane P, Froggatt P, Black T, Westgate J (1995). Chronology of Pliocene and Quaternary bioevents and climatic events from fission-track ages on tephra beds, Wairarapa, New Zealand. Earth Planet. Sci. Lett..

[8] Miller HBD, Vasconcelos PM, Eiler JM, Farley KA (2017). A Cenozoic terrestrial paleoclimate record from He dating and stable isotope geochemistry of goethites from Western Australia. Geology.

[9] Spiegel C, Kohn BP, Belton DX, Gleadow AJW (2007). Morphotectonic evolution of the central Kenya rift flanks: Implications for late Cenozoic environmental change in East Africa. Geology.

[10] Herman F (2013). Worldwide acceleration of mountain erosion under a cooling climate. Nature.

[11] Schneider, D. A. & Issler, D. R. Application of low-temperature thermochronology to hydrocarbon exploration. In *Fission-Track Thermochronology and its Application to Geology* 315–333 (Springer, 2019).

[12] Nixon AL (2022). Low-temperature thermal history of the McArthur Basin: Influence of the Cambrian Kalkarindji Large Igneous Province on hydrocarbon maturation. Basin Res..

[13] McInnes BIA, Evans NJ, Fu FQ, Garwin S (2005). Application of thermochronology to hydrothermal ore deposits. Rev. Mineral. Geochem..

[14] Glorie S, Hall JW, Nixon A, Collins AS, Reid A (2019). Carboniferous fault reactivation at the northern margin of the metal-rich Gawler Craton (South Australia): Implications for ore deposit exhumation and preservation. Ore Geol. Rev..

[15] Gong L (2021). Exhumation and preservation of paleozoic porphyry cu deposits: Insights from the Yandong deposit, Southern Central Asian Orogenic Belt. Econ. Geol..

[16] Sun, Y., Kohn, B. P., Boone, S. C., Wang, D. & Wang, K. Burial and exhumation history of the Lujing uranium ore field, Zhuguangshan, South China: Evidence from low-temperature thermochronology. *Minerals* (2021).

[17] Gorynski KE, Walker JD, Stockli DF, Sabin A (2014). Apatite (U-Th)/He thermochronometry as an innovative geothermal exploration tool: A case study from the southern Wassuk Range, Nevada. J. Volcanol. Geotherm. Res..

[18] Milesi G (2020). Tracking geothermal anomalies along a crustal fault using (U-Th)ĝ•He apatite thermochronology and rare-earth element (REE) analyses: The example of the Têt fault (Pyrenees, France). Solid Earth.

[19] Tagami T, Shimada C (1996). Natural long-term annealing of the zircon fission track system around a granitic pluton. J. Geophys. Res. Solid Earth.

[20] Danišík M (2012). Re-anchoring the late Pleistocene tephrochronology of New Zealand based on concordant radiocarbon ages and combined 238U/230Th disequilibrium and (U-Th)/He zircon ages. Earth Planet. Sci. Lett..

[21] Gleadow A, Harrison M, Kohn B, Lugo-zazueta R, Phillips D (2015). The Fish Canyon Tuff: A new look at an old low-temperature thermochronology standard. Earth Planet. Sci. Lett..

[22] Whipp DM, Ehlers TA (2007). Influence of groundwater flow on thermochronometer-derived exhumation rates in the central Nepalese Himalaya. Geology.

[23] Boone SC, Seiler C, Reid AJ, Kohn B, Gleadow A (2016). An Upper Cretaceous paleo-aquifer system in the Eromanga Basin of the central Gawler Craton, South Australia: Evidence from apatite fission track thermochronology. Aust. J. Earth Sci..

[24] Seiler C, Gleadow AJW, Fletcher JM, Kohn BP (2009). Thermal evolution of a sheared continental margin: Insights from the Ballenas transform in Baja California, Mexico. Earth Planet. Sci. Lett..

[25] Armstrong EM (2022). A multi-proxy approach using zircon (U-Th)/He thermochronometry and biomarker thermal maturity to robustly capture earthquake temperature rise along the punchbowl fault, California. Geochem. Geophys. Geosyst..

[26] d’Alessio MA, Blythe AE, Bürgmann R (2003). No frictional heat along the San Gabriel fault, California: Evidence from fission-track thermochronology. Geology.

[27] Mitchell SG, Reiners PW (2003). Influence of wildfires on apatite and zircon (U-Th)/He ages. Geology.

[28] Ganapathy R, Anders E (1969). Ages of calcium-rich achondrites—II. Howardites, nakhlites, and the Angra dos Reis angrite. Geochim. Cosmochim. Acta.

[29] Fleischer R, Price P, Walker R (1965). Ion explosion spike mechanism for formation of charged-particle tracks in solids. J. Appl. Phys..

[30] Naeser, C. W. Fission-track dating and geologic annealing of fission tracks. In *Lectures in isotope geology* 154–169 (Springer Berlin Heidelberg, 1979).

[31] Flowers RM (2022). (U-Th)/He chronology: Part 1. Data, uncertainty, and reporting. GSA Bull..

[32] Reiners PW (2017). Geochronology and Thermochronology.

[33] Wagner G, Van den Haute P (1992). Fission-Track Dating.

[34] Gleadow AJW, Belton DX, Kohn BP, Brown RW (2002). Fission track dating of phosphate minerals and the thermochronology of apatite. Phosphates Geochem. Geobiol. Mater. Importance.

[35] Barbarand J, Carter A, Wood I, Hurford T (2003). Compositional and structural control of fission-track annealing in apatite. Chem. Geol..

[36] Brown RW (2013). Natural age dispersion arising from the analysis of broken crystals. Part I: Theoretical basis and implications for the apatite (U-Th)/He thermochronometer. Geochim. Cosmochim. Acta.

[37] Farley, K. A. (U-Th)/He Dating: Techniques, calibrations, and applications. In *Noble Gases in Geochemistry and Cosmochemistry. Reviews in Mineralogy and Geochemistry. No.47.* 819–844 (Mineralogical Society of America, Washington, DC, 2002).

[38] Shuster DL, Flowers RM, Farley KA (2006). The influence of natural radiation damage on helium diffusion kinetics in apatite. Earth Planet. Sci. Lett..

[39] Carlson WD, Donelick RA, Ketcham RA (1999). Variability of apatite fission-track annealing kinetics: II. Crystallographic orientation effects. Am. Mineral..

[40] Donelick RA, O’Sullivan PB, Ketcham RA (2005). Apatite fission-track analysis. Rev. Mineral. Geochem..

[41] Ketcham RA, Carter A, Donelick RA, Barbarand J, Hurford AJ (2007). Improved modeling of fission-track annealing in apatite. Am. Mineral..

[42] Guenthner WR, Reiners PW, Ketcham RA, Nasdala L, Giester G (2013). Helium diffusion in natural zircon: radiation damage, anisotropy, and the interpretation of zircon (U-TH)/He thermochronology. Am. J. Sci..

[43] Braun J, van der Beek P, Batt G (2006). Quantitative Thermochronology: Numerical Methods for the Interpretation of Thermochronological data.

[44] Ketcham RA (2005). Forward and inverse modeling of low-temperature thermochronometry data. Rev. Mineral. Geochem..

[45] Gallagher K (2012). Transdimensional inverse thermal history modeling for quantitative thermochronology. J. Geophys. Res. Solid Earth.

[46] Boone SC (2022). AusGeochem: An open platform for geochemical data preservation, dissemination and synthesis. Geostand. Geoanal. Res..

[47] Wilkinson MD (2016). Comment: The FAIR Guiding Principles for scientific data management and stewardship. Sci. Data.

[48] Stall S (2019). Make scientific data FAIR. Nature.

[49] Sherratt T (2019). From portals to platforms: Building new frame works for user engagement. LIANZA.

[50] Jones, S. L., Anderson, J. R., Fraser, G. L., Lewis, C. J. & McLennan, S. M. *A U–Pb Geochronology Compilation for Northern Australia: Version 2, 2018. Record 2018/49.* (2018).

[51] Gleadow AJW (2009). Coincidence mapping – a key strategy for the automatic counting of fission tracks in natural minerals. Geol. Soc. London Spec. Publ. Thermochronological Methods From Palaeotemperature Constraints to Landsc. Evol. Model..

[52] McMillan, M. F., Boone, S. C., Kohn, B. P., Gleadow, A. J. & Chindandali, P. R. Development of the Nyika Plateau, Malawi: A long lived paleo-surface or a contemporary feature of the East African Rift? *Geochem. Geophys. Geosyst.* **23** (2022).

[53] Klump J (2021). Towards globally unique identification of physical samples: Governance and technical implementation of the IGSN global sample number. Data Sci. J..

[54] Merdith AS (2021). Extending full-plate tectonic models into deep time: Linking the Neoproterozoic and the Phanerozoic. Earth-Science Rev..

[55] Gleadow AJW, Kohn BP, Brown RW, O’Sullivan PB, Raza A (2002). Fission track thermotectonic imaging of the Australian continent. Tectonophysics.

[56] Dunkl I (2002). Trackkey: a Windows program for calculation and graphical presentation of fission track data. Comput. Geosci..

[57] Braun J (2012). Quantifying rates of landscape evolution and tectonic processes by thermochronology and numerical modeling of crustal heat transport using PECUBE. Tectonophysics.

[58] Müller RD (2018). GPlates: Building a virtual earth through deep time. Geochem. Geophys. Geosyst..

[59] Cao W (2017). Improving global paleogeography since the late Paleozoic using paleobiology. Biogeosciences.

[60] Moore ME, Gleadow AJW, Lovering JF (1986). Thermal evolution of rifted continental margins: New evidence from fission tracks in basement apatites from southeastern Australia. Earth Planet. Sci. Lett..

[61] van der Beek PA, Braun J, Lambeck K (1999). Post-Palaeozoic uplift history of southeastern Australia revisited: Results from a process-based model of landscape evolution. Aust. J. Earth Sci..

[62] Persano C, Stuart FM, Bishop P, Dempster TJ (2005). Deciphering continental breakup in eastern Australia using low-temperature thermochronometers. J. Geophys. Res. Solid Earth.

[63] Persano C, Stuart FM, Bishop P, Barfod D (2002). Apatite (U-Th)/He age constraints on the development of the Great Escarpment on the southeastern Australian passive margin. Earth Planet. Sci. Lett..

[64] Braun J, van der Beek P (2004). Evolution of passive margin escarpments: What can we learn from low-temperature thermochronology?. J. Geophys. Res..

[65] Wildman, M., Cogné, N. & Beucher, R. Fission-Track Thermochronology Applied to the Evolution of Passive Continental Margins. In *Fission-Track Thermochronology and its Application to Geology* 351–371 (Springer International Publishing, 2019). 10.1007/978-3-319-89421-8_20.

[66] Green P (1986). On the thermo-tectonic evolution of Northern England: evidence from fission track analysis. Geol. Mag..

[67] Gallagher K, Hawkesworth CJ, Mantovani MSM (1994). The denudation history of the onshore continental margin of SE Brazil inferred from apatite fission track data. J. Geophys. Res..

[68] Gallagher K, Brown R (1999). Denudation and uplift at passive margins: The record on the Atlantic Margin of southern Africa. Philos. Trans. R. Soc. A Math. Phys. Eng. Sci..

[69] Gunnell Y, Gallagher K, Carter A, Widdowson M, Hurford AJ (2003). Denudation history of the continental margin of western peninsular India since the early Mesozoic - reconciling apatite fission-track data with geomorphology. Earth Planet. Sci. Lett..

[70] Green PF, Lidmar-Bergström K, Japsen P, Bonow JM, Chalmers JA (2013). Stratigraphic landscape analysis, thermochronology and the episodic development of elevated, passive continental margins. Geol. Surv. Denmark Greenl. Bull..

[71] Green PF, Japsen P, Chalmers JA, Bonow JM, Duddy IR (2018). Post-breakup burial and exhumation of passive continental margins: Seven propositions to inform geodynamic models. Gondwana Res..

[72] McMillan M, Gleadow A, Kohn B, Seiler C (2020). Post Gondwana breakup evolution of the SE Australia rifted margin revisited. Terra Nova.

[73] Foster DA, Gleadow JW (1996). Structural framework and denudation history of the flanks of the Kenya and Anza Rifts, East Africa. Tectonics.

[74] Bosworth W (1992). Mesozoic and early Tertiary rift tectonics in East Africa. Tectonophysics.

[75] Boone SC (2018). Influence of rift superposition on lithospheric response to East African Rift System Extension: Lapur Range, Turkana, Kenya. Tectonics.

[76] Torres Acosta V (2015). Cenozoic extension in the Kenya Rift from low-temperature thermochronology: Links to diachronous spatiotemporal evolution of rifting in East Africa. Tectonics.

[77] Latin D, Norry MJ, Tarzey RJE (1993). Magmatism in the gregory rift, East Africa: Evidence for melt generation by a plume. J. Petrol..

[78] Rooney TO (2017). The Cenozoic magmatism of East-Africa: Part I—Flood basalts and pulsed magmatism. Lithos.

[79] Rooney TO (2020). The Cenozoic magmatism of East Africa: Part V—Magma sources and processes in the East African Rift. Lithos.

[80] BEICIP. Geological Map of Kenya (1987).

[81] Boone SC, Balestrieri ML, Kohn B (2021). Tectono-thermal evolution of the Red Sea Rift. Front. Earth Sci..

[82] Boone SC, Balestrieri ML, Kohn B (2021). Thermo-tectonic imaging of the Gulf of Aden-Red Sea rift systems and Afro-Arabian hinterland. Earth-Science Rev..

[83] Bosworth, W. Geological Evolution of the Red Sea: Historical Background, Review, and Synthesis. In *The Red Sea* 45–78 (2015). 10.1007/978-3-662-45201-1_3.

[84] Ghebreab W, Carter A, Hurford AJ, Jouniaux L (2002). Constraints for timing of extensional tectonics in the western margin of the Red Sea in Eritrea. Earth Planet. Sci. Lett..

[85] Stockli, D. F. & Bosworth, W. Timing of extensional faulting along the magma-poor central and northern Red Sea rift margin—transition from regional extension to necking along a hyperextended rifted margin. In *Geological setting, Palaeoenvironment and Archaeology of the Red Sea* 81–111 (Springer, 2018).

[86] Bohannon RG, Naeser CW, Schmidt DL, Zimmermann RA (1989). The timing of uplift, volcanism, and rifting peripheral to the Red Sea: A case for passive rifting?. J. Geophys. Res..

[87] Daradich A, Mitrovica JX, Pysklywec RN, Willett SD, Forte AM (2003). Mantle flow, dynamic topography, and rift-flank uplift of Arabia. Geology.

[88] Martin EL, Barrote VR, Cawood PA (2022). A resource for automated search and collation of geochemical datasets from journal supplements. Sci. Data.

[89] Naeser CW (1967). The use of apatite and sphene for fission track age determinations. Geol. Soc. Am. Bull..

[90] Hasebe N, Barbarand J, Jarvis K, Carter A, Hurford AJ (2004). Apatite fission-track chronometry using laser ablation ICP-MS. Chem. Geol..

[91] Chew DM, Donelick RA (2012). Combined apatite fission track and U-Pb dating by LA-ICP-MS and its application in apatite provenance analysis. Mineral. Assoc. Canada Short Course.

[92] Gombosi DJ, Garver JI, Baldwin SL (2014). On the development of electron microprobe zircon fission-track geochronology. Chem. Geol..

[93] Evans NJ (2015). An in situ technique for (U-Th-Sm)/He and U-Pb double dating. J. Anal. At. Spectrom..

[94] Danišík, M. Integration of Fission-Track Thermochronology with Other Geochronologic Methods on Single Crystals. In *Fission-Track Thermochronology and its Application to Geology* 93–108 (Springer, 2019). 10.1007/978-3-319-89421-8_5.

