# Supplementary material for: A geospatial platform for the tectonic interpretation of low-temperature thermochronology Big Data
Source: Sci Rep. 2023 May 26;13:8581. doi: 10.1038/s41598-023-35776-3 (PMC10219985; doi:10.1038/s41598-023-35776-3)
Supplement: Supplementary file 1 — Supplementary Information 1. [file 41598_2023_35776_MOESM1_ESM.docx]

Supplementary Information for

A geospatial platform for the tectonic interpretation of

low-temperature thermochronology Big Data

*Samuel C Boone^1,2^, Fabian Kohlmann^3^, Wayne Noble^3^, Moritz Theile^3^, Romain Beucher^4^, Barry Kohn^1^, Stijn Glorie^2^, Martin Danišík^5^, Renjie Zhou^6^, Malcolm McMillan^1^, Angus Nixon^2^, Andrew Gleadow^1^, Xiaodong Qin^7^, Dietmar Müller^7^, and Brent McInnes^5^

Presented below are the fission track (Tables S1-S5) and (U-Th)/He (Tables S6-S8) datatables for *AusGeochem*, including data type and unit specifications, and field descriptions. The (U-Th)/He data model and corresponding tables (Tables S6-S8) were designed following the data reporting best-practices agreed on by the international expert community^31^. While the fission-track model (Tables S1-S5) was designed after the recommended data reporting practices currently being prepared for the same *Geological Society of America Bulletin* paper on “Reporting and Interpretation of Fission-Track Chronology Data”, led by B.K. and with co-authors S.B, M.D., and A.G. also being involved.

Tables S9 and S10 list the references where the low-temperature thermochronology data used in the Kenyan (Figure 6) and Red Sea (Figures 7 and 8) compilations were sourced from.

**Table S1. (Meta-)data fields of the FT Data Point (Version 2.1.0), which record analytical metadata and fission track data on the rock sample scale. Blue fields are inherited from the *AusGeochem* Core Model^46^.**

| **Field** | **Datatype** | **Unit** | **Description** |
| --- | --- | --- | --- |
| Sample ID | String |  | ID of sample analysed, usually assigned by sample collector |
| IGSN | String |  | International Geo Sample Number |
| Associated Literature | List |  | Database assigned ID for any particular publication |
| Laboratory | List |  | Lab name and/or Uni where analysis was conducted |
| Analyst | List |  | ORCID ID of analyst |
| Analysis Date-Time | Time |  | Date-time of analysis |
| Mineral Type | List |  | Mineral type analysed |
| Reference Material | List |  | Name of secondary reference material. NOTE: Only to be populated when datapoint refers to fission track analysis of a secondary reference material. |
| Batch ID (if applicable) | String |  | ID of analytical batch, allowing related unknown and secondary reference material results to be linked. |
| FT Characterisation Method | List |  | Method used to count and characterise fission tracks |
| FT Analytical Software | List |  | Software used to perform digital fission track analysis |
| FT Analytical Algorithm | List |  | The algorithm used to perform (semi-) automated FT counting |
| FT U Determination Technique | List |  | Analytical method used to measure uranium concentrations for FT age determinations |
| Etchant | List |  | Etchant chemical composition |
| Etchant Time | Float | seconds | Duration of etching |
| Etchant Temperature | Float | Celsius degrees | Temperature minerals were etched at |
| Cf Irradiation Y/N? | Boolean |  | Was the sample irradiated with ^252^Cf? |
| No. of Grains | Integer |  | Total number of single grains analysed |
| Area | Float | cm^2^ | Total area of counting region |
| ρ_d_ | Float | cm^-2^ | Mean dosimeter track density |
| N_d_ | Integer |  | Total number of dosimeter tracks |
| ρ_s_ | Float | cm^-2^ | Mean spontaneous track density |
| N_s_ | Integer |  | Total number of spontaneous tracks |
| ρ_i_ | Float | cm^-2^ | Mean induced track density |
| N_i_ | Integer |  | Total number of induced tracks |
| Dosimeter | List |  | Dosimeter glass used for analysis (only relevant for EDM and population fission track methods) |
| Mean U Content | Float | ppm | Average U content of analysed grains |
| U Standard Deviation | Float | ppm | Standard deviation of average U content of analysed grains |
| Mean U/Ca Ratio | Float |  | Average U/Ca ratio of analysed grains |
| U/Ca Ratio Standard Deviation | Float |  | Standard deviation of average U/Ca ratio of analysed grains |
| Mean Dpar | Float | micrometres | Mean etch pit diameter parallel to crystallographic c-axis |
| Dpar Standard Error | Float | micrometres | Standard error of etch pit diameter parallel to crystallographic c-axis |
| Total Number of Dpar Measurements | Integer |  | The total number of Dpar measurements for the entire sample |
| Mean Dper | Float | micrometres | Mean etch pit diameter perpendicular to crystallographic c-axis |
| Dper Standard Error | Float | micrometres | Standard error of etch pit diameter perpendicular to crystallographic c-axis |
| Total Number of Dper Measurements | Integer |  | The total number of Dper measurements for the entire sample |
| Mean r_mr0_ | Float |  | Mean r_mr0_ of analysed grains, a parameter corresponding to annealing resistance of an apatite grain of certain composition^39,41^ |
| r_mr0_ Standard Deviation | Float |  | Standard deviation of r_mr0_ |
| r_mr0_ Equation | List |  | The equation used to determine the r_mr0_ and κ parameters |
| Mean κ | Float |  | Mean fitted parameter corresponding to annealing resistance of an apatite grain of certain composition^39,41^ |
| κ Standard Deviation | Float |  | Standard deviation of mean K parameter |
| FT Mean Age | Float | Ma | FT mean age |
| FT Mean Age Uncertainty | Float | Ma | FT mean age uncertainty |
| FT Central Age | Float | Ma | FT central age |
| FT Central Age Uncertainty | Float | Ma | FT central age uncertainty |
| FT Pooled Age | Float | Ma | FT pooled age |
| FT Pooled Age Uncertainty | Float | Ma | FT pooled age uncertainty |
| FT Population Age | Float | Ma | FT population age |
| FT Population Age Uncertainty | Float | Ma | FT population age uncertainty |
| Age Uncertainty Type | List |  | FT age uncertainty type |
| P(χ^2^) | Float | % | Chi-square test to statistically test the null-hypothesis that the analysed grains belong to one age population |
| Dispersion | Float |  | Measure of dispersion of single grain ages, ranging from 0 to 1 |
| FT Age Equation | List |  | The equation used to determine FT age |
| ζ Calibration | Float | yr cm^2^ | Zeta for EDM or LA-ICP-MS zeta-calibrated fission track ages |
| ζ Calibration Uncertainty | Float | yr cm^2^ | Zeta uncertainty for EDM or LA-ICP-MS zeta-calibrated fission track ages |
| ζ Uncertainty Type | List |  | Zeta-calibration uncertainty type |
| R | Float | micrometres | R is the etchable fission track range used for determination of FT age via absolute dating approach |
| λ | List |  | Total ^238^U decay constant used to determine FT age |
| λ_f_ | List |  | Fission decay constant used to determine FT age |
| q | Float |  | Detection efficiency factor |
| Irradiation Reactor | List |  | Name of irradiation reactor for EDM and population age determinations |
| Thermal Neutron Dose | Integer |  | Thermal neutron dose during sample irradiation (This parameter is only required for fission track ages determined using the Population Method) |
| MTL | Float | micrometres | **M**ean confined fission **T**rack **L**ength |
| No. Tracks | Integer |  | Number of tracks measured |
| MTL Standard Error | Float | micrometres | Standard error of mean confined track length |
| MTL Standard Deviation | Float | micrometres | Standard deviation of mean confined fission track length |
| Comment | Text |  | Additional information about analysis or data upload |

**Table S2. FT Single Grain data table.**

| **Field** | **Datatype** | **Unit** | **Description** |
| --- | --- | --- | --- |
| Mount ID (FT Count) | String |  | Name or ID of sample mount used for fission track counting |
| Grain ID | String |  | Name or lab number of individual grain analysed |
| U Content | Float | ppm | Uranium content of analysed grain |
| U Uncertainty | Float | ppm | Uncertainty of uranium content of analysed grain |
| U/Ca Ratio | Float |  | U/Ca ratio of analysed grain |
| U/Ca Ratio Uncertainty | Float |  | Uncertainty of U/Ca ratio of analysed grain |
| U Uncertainty Type | List |  | Uncertainty type |
| FT Age | Float | Ma | FT age |
| FT Age Uncertainty | Float | Ma | FT age uncertainty |
| Age Uncertainty Type | List |  | Uncertainty type |
| r_mr0_ | Float |  | Parameter corresponding to annealing resistance of an apatite grain of certain composition^39,41^ |
| κ | Float |  | Parameter corresponding to annealing resistance of an apatite grain of certain composition^39,41^ |
| Comment | Text |  | Additional information about analysis or data upload |

**Table S3. FT Count Data table. In addition to manual line-by-line and bulk uploading via the AusGeochem csv template, users can directly upload count data from *FastTracks^51^* using FastTracks’s AusGeochemCount export data format.**

| **Field** | **Datatype** | **Unit** | **Description** |
| --- | --- | --- | --- |
| Grain ID | String |  | Name or lab number of individual grain analysed |
| Area | Float | cm^2^ | Total area of counting region |
| ρ_s_ | Float | cm^-2^ | Spontaneous track density |
| N_s_ | Integer |  | Number of spontaneous tracks |
| ρ_i_ | Float | cm^-2^ | Induced track density |
| N_i_ | Integer |  | Number of induced tracks |
| Dpar | Float | micrometres | Modal etch pit diameter parallel to crystallographic c-axis |
| Dpar Uncertainty | Float | micrometres | Uncertainty of etch pit diameter parallel to crystallographic c-axis |
| Number of Dpar Measurements | Integer |  | Number of Dpar measurements |
| Dper | Float | micrometres | Modal etch pit diameter perpendicular to crystallographic c-axis |
| Dper Uncertainty | Float | micrometres | Uncertainty of etch pit diameter perpendicular to crystallographic c-axis |
| Number of Dper Measurements | Integer |  | Number of Dper measurements |
| Uncertainty Type | List |  | Dpar and Dper measurement uncertainty type |
| Comment | Text |  | Additional information about analysis or data upload |

**Table S4. FT Length Data table. In addition to manual line-by-line and bulk uploading via the AusGeochem csv template, users can directly upload length data from *FastTracks*^51^ using FastTracks’s AusGeochemLength export data format.**

| **Field** | **Datatype** | **Unit** | **Description** |
| --- | --- | --- | --- |
| Mount ID (FT Lengths) | String |  | Name or ID of sample mount used for fission track length measurements |
| Etchant Time | Float | seconds | Duration of etching |
| Grain ID | String |  | Name or lab number of individual grain analysed |
| Track ID | String |  | The name or lab number of the measured track |
| Track Type | List |  | Type of track measurement (e.g., semi-track, confined track-in-track, confined track-in-cleavage) |
| Apparent Length | Float | micrometres | Apparent length measured parallel to grain surface |
| Corrected z-Depth | Float | micrometres | Distance in z-direction between track end points, corrected for refractive index of analysed mineral |
| Track Length | Float | micrometres | Fission track length |
| Azimuth | Float | degrees | Azimuth of track |
| Dip | Float | degrees | Dip of track |
| c-Axis Angle | Float | degrees | Angle of fission track to crystallographic c-axis |
| c-Axis Angle Corrected Length | Float | micrometres | c-axis corrected fission track length |
| Dpar | Float | micrometres | Modal etch pit diameter parallel to crystallographic c-axis |
| Dpar Uncertainty | Float | micrometres | Uncertainty of etch pit diameter parallel to crystallographic c-axis |
| Number of Dpar Measurements | Integer |  | The number of Dpar measurements |
| Dper | Float | micrometres | Modal etch pit diameter perpendicular to crystallographic c-axis |
| Dper Uncertainty | Float | micrometres | Uncertainty of etch pit diameter perpendicular to crystallographic c-axis |
| Number of Dper Measurements | Integer |  | The number of Dper measurements |
| Uncertainty Type | List |  | Dpar and Dper measurement uncertainty type |
| r_mr0_ | Float |  | Parameter corresponding to annealing resistance of an apatite grain of certain composition^39,41^ |
| κ | Float |  | Fitted parameter corresponding to annealing resistance of an apatite grain of certain composition^39,41^ |
| Comment | Text |  | Additional information about analysis or data upload |

**Table S5. FT Binned Length Data table, which is designed to accommodate the uploading of legacy data in cases where only confined track length histograms were provided in either one or two micron bins.**

| **Field** | **Datatype** | **Unit** | **Description** |
| --- | --- | --- | --- |
| Mount ID (FT Lengths) | String |  | Name or ID of sample mount used for fission track length measurements |
| Etchant Time | Float | seconds | Duration of etching |
| Dpar | Float | micrometres | Modal etch pit diameter parallel to crystallographic c-axis |
| Dpar Uncertainty | Float | micrometres | Uncertainty of etch pit diameter parallel to crystallographic c-axis |
| Number of Dpar Measurements | Integer |  | The number of Dpar measurements |
| Dper | Float | micrometres | Modal etch pit diameter perpendicular to crystallographic c-axis |
| Dper Uncertainty | Float | micrometres | Uncertainty of etch pit diameter perpendicular to crystallographic c-axis |
| Number of Dper Measurements | Integer |  | The number of Dper measurements |
| Uncertainty Type | List |  | Dpar and Dper measurement uncertainty type |
| 0-1 μm Bin | Integer |  | Number of measured confined tracks in 0 to 1 micron bin |
| 1-2 μm Bin | Integer |  | Number of measured confined tracks in 1 to 2 micron bin |
| 2-3 μm Bin | Integer |  | Number of measured confined tracks in 2 to 3 micron bin |
| 3-4 μm Bin | Integer |  | Number of measured confined tracks in 3 to 4 micron bin |
| 4-5 μm Bin | Integer |  | Number of measured confined tracks in 4 to 5 micron bin |
| 5-6 μm Bin | Integer |  | Number of measured confined tracks in 5 to 6 micron bin |
| 6-7 μm Bin | Integer |  | Number of measured confined tracks in 6 to 7 micron bin |
| 7-8 μm Bin | Integer |  | Number of measured confined tracks in 7 to 8 micron bin |
| 8-9 μm Bin | Integer |  | Number of measured confined tracks in 8 to 9 micron bin |
| 9-10 μm Bin | Integer |  | Number of measured confined tracks in 9 to 10 micron bin |
| 10-11 μm Bin | Integer |  | Number of measured confined tracks in 10 to 11 micron bin |
| 11-12 μm Bin | Integer |  | Number of measured confined tracks in 11 to 12 micron bin |
| 12-13 μm Bin | Integer |  | Number of measured confined tracks in 12 to 13 micron bin |
| 13-14 μm Bin | Integer |  | Number of measured confined tracks in 13 to 14 micron bin |
| 14-15 μm Bin | Integer |  | Number of measured confined tracks in 14 to 15 micron bin |
| 15-16 μm Bin | Integer |  | Number of measured confined tracks in 15 to 16 micron bin |
| 16-17 μm Bin | Integer |  | Number of measured confined tracks in 16 to 17 micron bin |
| 17-18 μm Bin | Integer |  | Number of measured confined tracks in 17 to 18 micron bin |
| 18-19 μm Bin | Integer |  | Number of measured confined tracks in 18 to 19 micron bin |
| 19-20 μm Bin | Integer |  | Number of measured confined tracks in 19 to 29 micron bin |
| Comment | Text |  | Additional information about analysis or data upload |

**Table S6. (Meta-)data fields of the He Data Point, which record analytical metadata and (U-Th)/He data on the rock sample scale. Blue fields are inherited from the *AusGeochem* Core Model^46^.**

| **Field** | **Datatype** | **Unit** | **Description** |
| --- | --- | --- | --- |
| Sample ID | String |  | ID of sample analysed, usually assigned by sample collector |
| IGSN | String |  | International Geo Sample Number |
| Associated Literature | List |  | Database assigned ID for any particular publication |
| Laboratory | List |  | Lab name and/or Uni where analysis was conducted |
| Analyst | List |  | ORCID ID of analyst |
| Analysis Date-Time | Time |  | Date-time of analysis |
| Mineral Type | List |  | Mineral type analysed |
| Mount ID (if appropriate) | String |  | Name or ID of sample mount used for in-situ (U-Th)/He analysis |
| Reference Material | List |  | Name of secondary reference material. NOTE: Only to be populated when datapoint refers to (U-Th)/He analysis of a secondary reference material. |
| Batch ID (if applicable) | String |  | ID of analytical batch, allowing related unknown and secondary reference material results to be linked. |
| Number of Aliquots | Integer |  | Number of aliquots analysed |
| Mean Uncorrected He Age | Float | Ma | Mean uncorrected He age |
| Mean Uncorrected He Age Uncertainty | Float | Ma | Uncertainty of mean uncorrected He age |
| Mean Uncorrected Age Uncertainty Type | List |  | Mean Uncorrected Age Uncertainty Type |
| Weighted Uncorrected Mean He Age | Float | Ma | Weighted mean uncorrected He age |
| Weighted Uncorrected Mean He Age Uncertainty | Float | Ma | Uncertainty of weighted mean uncorrected He age |
| Weighted Uncorrected Mean Age Uncertainty Type | List |  | Weighted Uncorrected Mean Age Uncertainty Type |
| MSWD of Weighted Mean Uncorrected Age | Float |  | Mean Square Weighted Deviation for reported weighted mean uncorrected He age |
| Weighted Mean Uncorrected Age 95% Confidence Interval | Float | Ma | The 95% confidence interval of the weighted mean uncorrected age |
| Weighted Mean Uncorrected Age P(χ^2) | Float | Percentage | Chi-squared test to statistically test the null-hypothesis that the analysed aliquots belong to one age population |
| Uncorrected Age Interquartile Range (IQR) | Float | Ma | Interquartile range (IQR) of aliquot He ages, a robust measure of statistical dispersion in intrasample He datasets^95^. |
| Mean Corrected He Age | Float | Ma | Mean corrected He age |
| Mean Corrected He Age Uncertainty | Float | Ma | Uncertainty of mean corrected He age |
| Uncertainty Type | List |  | Uncertainty type |
| Weighted Mean Corrected He Age | Float | Ma | Weighted mean corrected He age |
| Weighted Mean Corrected He Age Uncertainty | Float | Ma | Uncertainty of weighted mean corrected He age |
| Uncertainty Type | List |  | Uncertainty type |
| MSWD of Weighted Mean Corrected Age | Float |  | Mean Square Weighted Deviation for reported weighted mean corrected He age |
| Weighted Mean Corrected Age 95% Confidence Interval | Float | Ma | The 95% confidence interval of the weighted mean corrected age |
| Weighted Mean Corrected Age P(χ^2) | Float | Percentage | Chi-square test to statistically test the null-hypothesis that the analysed aliquots belong to one age population |
| Corrected Age Interquartile Range (IQR) | Float | Ma | Interquartile range (IQR) of aliquot He ages, a robust measure of statistical dispersion in intrasample He datasets^95^. |
| Uncertainty Factors Comment | String |  | Describe the factors included in uncertainty calculations (e.g., propagated precision on repeat measurements of the sample, blanks, spikes, and reference materials). See Section 7 in Flowers et al.^31^ for guidance. |
| Ablation Pit Measuring Technique | List |  | The method used to measure ablation pit volumes. Only applicable for in-situ (U-Th)/He analysis. |
| Pit Volume Determination Software | List |  | The software used to calculate ablation pit volumes. Only applicable for in-situ (U-Th)/He analysis. |
| In-Situ He Measurement Technique | List |  | The method used to measure 4He content in-situ. Only applicable for in-situ (U-Th)/He analysis. |
| In-Situ Parent Isotope Measurement Technique | List |  | The method used to measure parent isotope content in-situ. Only applicable for in-situ (U-Th)/He analysis. |
| Grain Dimensions Equations Reference | List |  | Reference to equations used for calculating surface area and volume of grains |
| Alpha Stopping Distances Reference | List |  | Reference to alpha stopping distances used in calculations |
| FT Equation | List |  | Alpha-ejection correction (FT) equation used |
| Rsv Equation | List |  | Equation used to calculate an equivalent spherical radius using the equivalent surface area to volume ratio approach |
| Rft Equation | List |  | Equation used to calculate an equivalent spherical radius using the radius of a sphere with an equivalent FT correction |
| eU Equation | List |  | Equation used to calculate eU concentration |
| He Age Equation | List |  | Approach employed to calculate the reported He age |
| Corrected He Age Determination Method | List |  | The method used to calculate the corrected He age |
| Comment | Text |  | Additional information about analysis or data upload |

**Table S7. He Whole Grain data table.**

| **Field** | **Datatype** | **Unit** | **Description** |
| --- | --- | --- | --- |
| Aliquot ID | String |  | Name or lab number of analysed aliquot (if available) |
| Aliquot Type | List |  | The type of aliquot analysed, i.e., single-grain, multi-grain or unknown |
| Number of Aliquot Grains | Integer |  | Number of grains in aliquot (only relevant for multi-grain aliquots) |
| Crystal Fragmentation | List |  | Is the crystal whole, fragmented, abraded, mixed (in the case of multi-grain aliquots) or unknown? |
| Aliquot Morphology | List |  | Morphology of analysed aliquot grain(s) |
| Assumed Aliquot Geometry | List |  | Assumed geometry of grain(s) |
| Aliquot Length | Float | μm | Length of analysed grain(s) (average value for multi-grain aliquots) |
| Average Aliquot Length Standard Deviation | Float | μm | One standard deviation of average length of analysed grains (only relevant for multi-grain aliquots) |
| Aliquot Width | Float | μm | Width of analysed grain(s) (average value for multi-grain aliquots) |
| Average Aliquot Width Standard Deviation | Float | μm | One standard deviation of average width of analysed grains (only relevant for multi-grain aliquots) |
| Aliquot Height | Float | μm | Height (second width) of analysed grain(s), if measured (average value for multi-grain aliquots) |
| Average Aliquot Height Standard Deviation | Float | μm | One standard deviation of average height (second width) of analysed grains, if measured (only relevant for multi-grain aliquots) |
| Surface Area | Float | μm^2^ | Surface area of analysed grain(s) (average value for multi-grain aliquots) |
| Average Surface Area Standard Deviation | Float | μm^2^ | One standard deviation of average surface area of analysed grains (only relevant for multi-grain aliquots) |
| Volume | Float | μm^3^ | Estimated volume of analysed grain(s) (average value for multi-grain aliquots) |
| Average Volume Standard Deviation | Float | μm^3^ | One standard deviation of average estimated volume of analysed grains (only relevant for multi-grain aliquots) |
| Pyramidal Termination Height 1 | Float | μm | Pyramidal termination 1 height of analysed grain(s), if measured (average value for multi-grain aliquots) |
| Average Pyramidal Termination Height 1 Standard Deviation | Float | μm | One standard deviation of average pyramidal termination 1 height of analysed grains, if measured (only relevant for multi-grain aliquots) |
| Pyramidal Termination Height 2 | Float | μm | Pyramidal termination 2 height of analysed grain(s), if measured (average value for multi-grain aliquots) |
| Average Pyramidal Termination Height 2 Standard Deviation | Float | μm | One standard deviation of average pyramidal termination 2 height of analysed grains, if measured (only relevant for multi-grain aliquots) |
| V/S Ratio | Float |  | Volume to surface area ratio of analysed grain(s); see eq. 15 in Hourigan et al.^96^ |
| F_T_ | Float |  | Mass-weighted mean alpha ejection correction (FT) of aliquot |
| F_T_ Uncertainty | Float |  | Estimated Uncertainty of alpha ejection correction. E.g., Farley et al.^97^ recommended an estimated FT error of 5% for FT > 0.6, 10% for FT < 0.6, 15% for FT < 0.5 |
| Uncertainty Type | List |  | Uncertainty type |
| R_SV_ | Float | μm | Average equivalent spherical radius of analysed aliquot, computed as the radius of a sphere with an equivalent surface area to volume ratio as analysed aliquot |
| R_FT_ | Float | μm | Average equivalent spherical radius of analysed aliquot, computed as the radius of a sphere with an equivalent FT correction |
| Assumed Mineral Density | Float | g/cm^3^ | Assumed mineral density used to determine mass using the dimensional approach |
| Ca Content | Float | ng | Total Ca content of aliquot in nanograms, if measured. Usually only measured if aliquot mass is determined stoichiometrically. |
| Ca Content Uncertainty | Float | ng | Total Ca content uncertainty, if measured. Usually only measured if aliquot mass is determined stoichiometrically. |
| Uncertainty Type | List |  | Uncertainty type |
| Zr Content | Float | ng | Total Zr content of aliquot in nanograms, if measured. Usually only measured if aliquot mass is determined stoichiometrically. |
| Zr Content Uncertainty | Float | ng | Total Zr content uncertainty, if measured. Usually only measured if aliquot mass is determined stoichiometrically. |
| Uncertainty Type | List |  | Uncertainty type |
| Assumed Mineral Chemical Formula | Float |  | Assumed chemical formula for mineral used to determine mass using the stoichiometric approach (e.g., for apatite, Ca_5_(PO_4_)_3_(OH) or Ca_5_(PO_4_)_3_F or Ca_5_(PO_4_)_3_Cl. |
| Estimated Aliquot Mass | Float | mg | Estimated aliquot mass |
| Estimated Aliquot Mass Uncertainty | Float | mg | Uncertainty of estimated aliquot mass |
| Uncertainty Type | List |  | Uncertainty type |
| ^4^He Absolute Amount | Float | ncc | Absolute He content of aliquot |
| ^4^He Absolute Amount Uncertainty | Float | ncc | Uncertainty of absolute He content of aliquot |
| Uncertainty Type | List |  | Uncertainty type |
| ^4^He Concentration | Float | nmol/g | ^4^He concentration of aliquot |
| ^4^He Concentration Uncertainty | Float | nmol/g | ^4^He concentration uncertainty of aliquot |
| Uncertainty Type | List |  | Uncertainty type |
| U Absolute Amount | Float | ng | Absolute amount of Uranium (^238^U + ^235^U) in aliquot |
| U Absolute Amount Uncertainty | Float | ng | Uncertainty of absolute amount of Uranium (^238^U + ^235^U) in aliquot |
| Uncertainty Type | List |  | Uncertainty type |
| U Concentration | Float | ppm | Uranium (^238^U + ^235^U) concentration of aliquot, calculated as μg/g |
| U Concentration Uncertainty | Float | ppm | Uncertainty of Uranium (^238^U + ^235^U) concentration of aliquot, calculated as μg/g |
| Uncertainty Type | List |  | Uncertainty type |
| Th Absolute Amount | Float | ng | Absolute amount of Thorium (^232^Th) in aliquot |
| Th Absolute Amount Uncertainty | Float | ng | Uncertainty of absolute amount of Thorium (^232^Th) in aliquot |
| Uncertainty Type | List |  | Uncertainty type |
| Th Concentration [ppm] | Float | ppm | Thorium (^232^Th) concentration of aliquot, calculated as μg/g |
| Th Concentration Uncertainty [ppm] | Float | ppm | Uncertainty of Thorium (^232^Th) concentration of aliquot, calculated as μg/g |
| Uncertainty Type | List |  | Uncertainty type |
| Sm Absolute Amount | Float | ng | Absolute amount of Sumerium (^147^Sm) in aliquot |
| Sm Absolute Amount Uncertainty | Float | ng | Uncertainty of absolute amount of Sumerium (^147^Sm) in aliquot |
| Uncertainty Type | List |  | Uncertainty type |
| Sm Concentration | Float | ppm | Sumerium (^147^Sm) concentration of aliquot, calculated as μg/g |
| Sm Concentration Uncertainty | Float | ppm | Uncertainty of Sumerium (^147^Sm) concentration of aliquot, calculated as μg/g |
| Uncertainty Type | List |  | Uncertainty type |
| Th/U | Float |  | Thorium/Uranium ratio |
| eU | Float | ppm | Effective uranium concentration |
| eU Uncertainty | Float | ppm | Effective uranium concentration uncertainty |
| Uncertainty Type | List |  | Uncertainty type |
| Uncorrected He Age | Float | Ma | Uncorrected He age |
| Uncorrected He Age Uncertainty | Float | Ma | Total analytical uncertainty on uncorrected He age (before FT correction). This should include the propagated uncertainties on the absolute amounts of daughter and parent. See Section 7 in Flowers et al.^31^ for guidance. |
| Uncertainty Type | List |  | Uncertainty type |
| Corrected He Age | Float | Ma | FT corrected He age |
| Total Analytical Uncertainty (Corrected Age) | Float | Ma | Total analytical uncertainty on corrected He age (FT corrected). This should include the propagated uncertainties on the absolute amounts of daughter and parent. See Section 7 in Flowers et al.^31^ for guidance. |
| Uncertainty Type | List |  | Uncertainty type |
| Total Analytical Uncertainty + FT (Corrected Age) | Float | Ma | Total analytical uncertainty on corrected He age (FT corrected) including an estimated uncertainty for Ft correction. This should include the propagated uncertainties on the absolute amounts of daughter and parent. See Section 7 in Flowers et al.^31^ for guidance. |
| Uncertainty Type | List |  | Uncertainty type |
| Comment | Text |  | Additional information about analysis or data upload |

**Table S8. He In-Situ data table.**

| **Field** | **Datatype** | **Unit** | **Description** |
| --- | --- | --- | --- |
| Grain ID | String |  | Name or lab number of analysed grain (if available) |
| Pit ID | String |  | Identifier for analysed pit |
| Crystal Fragmentation | List |  | Is the crystal whole, fragmented, abraded, or unknown? |
| He Measurement Pit Volume | Float | μm^3^ | Volume of ablation pit for He content measurement |
| He Measurement Pit Volume Uncertainty | Float | μm^3^ | Uncertainty of ablation pit volume for He content measurement |
| Uncertainty Type | List |  | Uncertainty type |
| Parent Isotopes Measurement Pit Volume | Float | μm^3^ | Volume of ablation pit for parent isotopic content measurement (if measured) |
| Parent Isotopes Measurement Pit Volume Uncertainty | Float | μm^3^ | Uncertainty of ablation pit volume for parent isotopic content measurement (if measured) |
| Uncertainty Type | List |  | Uncertainty type |
| ^4^He Absolute Amount | Float | ncc | Absolute He content |
| ^4^He Absolute Amount Uncertainty | Float | ncc | Uncertainty of absolute He content |
| Uncertainty Type | List |  | Uncertainty type |
| ^4^He Concentration | Float | nmol/g | ^4^He concentration |
| ^4^He Concentration Uncertainty | Float | nmol/g | ^4^He concentration uncertainty |
| Uncertainty Type | List |  | Uncertainty type |
| U Absolute Amount | Float | ng | Absolute amount of Uranium (^238^U + ^235^U) |
| U Absolute Amount Uncertainty | Float | ng | Uncertainty of absolute amount of Uranium (^238^U + ^235^U) |
| Uncertainty Type | List |  | Uncertainty type |
| U Concentration | Float | ppm | Uranium (^238^U + ^235^U) concentration, calculated as μg/g |
| U Concentration Uncertainty | Float | ppm | Uncertainty of Uranium (^238^U + ^235^U) concentration, calculated as μg/g |
| Uncertainty Type | List |  | Uncertainty type |
| Th Absolute Amount | Float | ng | Absolute amount of Thorium (^232^Th) |
| Th Absolute Amount Uncertainty | Float | ng | Uncertainty of absolute amount of Thorium (^232^Th) |
| Uncertainty Type | List |  | Uncertainty type |
| Th Concentration [ppm] | Float | ppm | Thorium (^232^Th) concentration, calculated as μg/g |
| Th Concentration Uncertainty [ppm] | Float | ppm | Uncertainty of Thorium (^232^Th) concentration, calculated as μg/g |
| Uncertainty Type | List |  | Uncertainty type |
| Sm Absolute Amount | Float | ng | Absolute amount of Samarium (^147^Sm) |
| Sm Absolute Amount Uncertainty | Float | ng | Uncertainty of absolute amount of Samarium (^147^Sm) |
| Uncertainty Type | List |  | Uncertainty type |
| Sm Concentration | Float | ppm | Samarium (^147^Sm) concentration, calculated as μg/g |
| Sm Concentration Uncertainty | Float | ppm | Uncertainty of Samarium (^147^Sm) concentration, calculated as μg/g |
| Uncertainty Type | List |  | Uncertainty type |
| eU | Float | ppm | Effective uranium concentration |
| eU Uncertainty | Float | ppm | Effective uranium concentration uncertainty |
| Uncertainty Type | List |  | Uncertainty type |
| Relationship of He and Parent Isotopes Measurement Locations | List |  | The spatial relationship between the He and parent isotope measurement beam locations/ablation pits |
| Uncorrected He Age | Float | Ma | Uncorrected He age |
| Uncorrected He Age Uncertainty | Float | Ma | Total analytical uncertainty on uncorrected He age (before FT correction). This should include the propagated uncertainties on the absolute amounts of daughter and parent. See Section 7 in Flowers et al.^31^ for guidance. |
| Uncertainty Type | List |  | Uncertainty type |
| Age Calibration Factor (if applicable) | Float |  | He Age Calibration factor, if applicable (following the methods of Pickering et al.^98^) |
| Calibration Factor Corrected He Age (if applicable) | Float | Ma | Calibration factor corrected He age (if calculated) |
| Calibration Factor Corrected He Age Uncertainty (if applicable) | Float | Ma | Total analytical uncertainty on corrected He age (FT corrected). This should include the propagated uncertainties on the absolute amounts of daughter and parent. See Section 7 in Flowers et al.^31^ for guidance. |
| Uncertainty Type | List |  | Uncertainty type |
| Comment | Text |  | Additional information about analysis or data upload |

# References

31. Flowers, R. M. *et al.* (U-Th)/He chronology: Part 1. Data, uncertainty, and reporting. *GSA Bull.* 1–33 (2022) doi:10.1130/b36266.1.

39. Carlson, W. D., Donelick, R. A. & Ketcham, R. A. Variability of apatite fission-track annealing kinetics: II. Crystallographic orientation effects. *Am. Mineral.* **84**, 1224–1234 (1999).

41. Ketcham, R. A., Carter, A., Donelick, R. A., Barbarand, J. & Hurford, A. J. Improved modeling of fission-track annealing in apatite. *Am. Mineral.* **92**, 799–810 (2007).

46. Boone, S. C. *et al.* AusGeochem: An Open Platform for Geochemical Data Preservation, Dissemination and Synthesis. *Geostand. Geoanalytical Res.* (2022) doi:10.1111/ggr.12419.

51. Gleadow, A. J. W. *et al.* Coincidence mapping – a key strategy for the automatic counting of fission tracks in natural minerals. *Geol. Soc. London Spec. Publ. Thermochronological Methods From Palaeotemperature Constraints to Landsc. Evol. Model.* **324**, 25–36 (2009).

95. Boone, S. C., Balestrieri, M. L., Kohn, B. P., Corti, G., Gleadow, A. J. W., & Seiler, C. (2019). Tectonothermal evolution of the broadly rifted zone, Ethiopian Rift. Tectonics, 38(3), 1070–1100. https://doi.org/10.1029/2018TC005210

96. J. K. Hourigan, P. W. Reiners, M. T. Brandon, U-Th zonation-dependent alpha-ejection in (U-Th)/He chronometry. *Geochim. Cosmochim. Acta*. **69**, 3349–3365 (2005).

97. K. A. Farley, R. A. Wolf, L. T. Silver, The effects of long alpha-stopping distances on (U-Th)/He ages. *Geochim. Cosmochim. Acta*. **60**, 4223–4229 (1996).

98. J. Pickering, W. Matthews, E. Enkelmann, B. Guest, C. Sykes, B. M. Koblinger, Laser ablation (U-Th-Sm)/He dating of detrital apatite. *Chem. Geol.* **548**, 119683 (2020).

99. S. C. Boone, C. Seiler, B. P. Kohn, A. J. W. Gleadow, D. A. Foster, L. Chung, Influence of Rift Superposition on Lithospheric Response to East African Rift System Extension: Lapur Range, Turkana, Kenya. *Tectonics* (2018), doi:10.1002/2017TC004575.

100. S. C. Boone, B. P. Kohn, A. J. W. Gleadow, C. K. Morley, C. Seiler, D. A. Foster, L. Chung, Tectono-thermal evolution of a long-lived segment of the East African Rift System: Thermochronological insights from the North Lokichar Basin, Turkana, Kenya. *Tectonophysics*. **744**, 23–46 (2018).

101. S. C. Boone, B. P. Kohn, A. J. W. Gleadow, C. K. Morley, C. Seiler, D. A. Foster, Birth of the East African Rift System: Nucleation of magmatism and strain in the Turkana Depression. *Geology*. **47** (2019), doi:10.1130/G46468.1.

102. D. A. Foster, A. J. W. Gleadow, The morphotectonic evolution of rift-margin mountains in central Kenya: Constraints from apatite fission-track thermochronology. *Earth Planet. Sci. Lett.* **113**, 157–171 (1992).

103. A. J. W. Gleadow, Fission track age of the KBS Tuff and associated hominid remains in northern Kenya. *Nature*. **284**, 225–230 (1980).

104. W. P. Noble, thesis, La Trobe University (1997).

105. D. A. Majer-Kielbaska, thesis, University of Melbourne (2015).

106. M. Wagner, R. Altherr, P. Van Den Haute, Apatite fission-track analysis of Kenyan basement rocks: constraints on the thermotectonic evolution of the Kenya dome. A reconnaissance study. *Tectonophysics*. **204**, 93–110 (1992).

107. E. Abbate, M. L. Balestrieri, G. Bigazzi, Morphostructural development of the Eritrean rift flank (southern Red Sea) inferred from apatite fission track analysis. *J. Geophys. Res.* **107**, 1–12 (2002).

108. B. P. Kohn, S. Feinstein, D. A. Foster, M. S. Steckler, M. Eyal, Thermal history of the eastern Gulf of Suez, II. Reconstruction from apatite fission track and 40Ar/39Ar K-feldspar measurements. *Tectonophysics*. **283**, 219–239 (1997).

109. B. Kohn, T. Weissbrod, L. Chung, K. Farley, S. Bodorkos, Low-temperature thermochronology of francolite: Insights into timing of Dead Sea Transform motion. *Terra Nov.* **31**, 205–219 (2019).

110. M. A. Menzies, J. Baker, D. Bosence, C. Dart, I. Davison, A. Hurford, M. Al’Kadasi, K. McClay, G. Nichols, A. Al’Subbary, A. Yelland, The timing of magmatism, uplift and crustal extension: Preliminary observations from Yemen. *Geol. Soc. Spec. Publ.* **68**, 293–304 (1992).

111. M. Menzies, K. Gallagher, A. Yelland, A. J. Hurford, Volcanic and nonvolcanic rifted margins of the Red Sea and Gulf of Aden: Crustal cooling and margin evolution in Yemen. *Geochim. Cosmochim. Acta*. **61**, 2511–2527 (1997).

112. N. Morag, I. Haviv, M. Eyal, B. P. Kohn, S. Feinstein, Early flank uplift along the Suez Rift: Implications for the role of mantle plumes and the onset of the Dead Sea Transform. *Earth Planet. Sci. Lett.* **516**, 56–65 (2019).

113. D. Naylor, M. Al-Rawi, G. Clayton, M. J. Fitzpatrick, P. F. Green, Hydrocarbon potential in Jordan. *J. Pet. Geol.* **36**, 205–236 (2013).

114. G. I. Omar, B. P. Kohn, T. M. Lutz, H. Faul, The cooling history of Silurian to Cretaceous alkaline ring complexes, south Eastern Desert, Egypt, as revealed by fission-track analysis. *Earth Planet. Sci. Lett.* **83**, 94–108 (1987).

115. G. I. Omar, M. S. Steckler, W. R. Buck, B. P. Kohn, Fission-track analysis of basement apatites at the western margin of the Gulf of Suez rift, Egypt: evidence for synchroneity of uplift and subsidence. *Earth Planet. Sci. Lett.* **94**, 316–328 (1989).

116. E. Szymanski, D. F. Stockli, P. R. Johnson, C. Hager, Thermochronometric evidence for diffuse extension and two-phase rifting within the Central Arabian Margin of the Red Sea Rift. *Tectonics*. **35**, 2863–2895 (2016).

117. P. Vermeesch, D. Avigad, M. O. McWilliams, 500 Myr of thermal history elucidated by multi-method detrital thermochronology of North Gondwana Cambrian sandstone (Eilat area, Israel). *Bull. Geol. Soc. Am.* **121**, 1204–1216 (2009).

118. E. Abbate, M. L. Balestrieri, G. Bigazzi, Uplifted rift-shoulder of the Gulf of Aden in northwestern Somalia: Palinspastic reconstructions supported by apatite fission-track data. *Mémoires du Muséum Natl. d’histoire Nat.* **186**, 629–640 (2001).

119. M. L. Balestrieri, F. M. Stuart, C. Persano, E. Abbate, G. Bigazzi, Geomorphic development of the escarpment of the Eritrean margin, southern Red Sea from combined apatite fission-track and (U–Th)/He thermochronometry. *Earth Planet. Sci. Lett.* **231**, 97–110 (2005).

120. T. Abebe, M. L. Balestrieri, G. Bigazzi, The Central Main Ethiopian Rift is younger than 8 Ma: confirmation through apatite fission-track thermochronology. *Terra Nov.* **22**, 470–476 (2010).

121. M. L. Balestrieri, E. Abbate, G. Bigazzi, O. E. B. Ali, Thermochronological data from Sudan in the frame of the denudational history of the Nubian Red Sea margin. *Earth Surf. Process. Landforms*. **34**, 1279–1290 (2009).

122. A. V. Bojar, H. Fritz, S. Kargl, W. Unzog, Phanerozoic tectonothermal history of the Arabian-Nubian shield in the Eastern Desert of Egypt: Evidence from fission track and paleostress data. *J. African Earth Sci.* **34**, 191–202 (2002).

123. S. Feinstein, M. Eyal, B. P. Kohn, M. S. Steckler, K. M. Ibrahim, B. K. Moh’d, Y. Tian, Uplift and denudation history of the eastern Dead Sea rift fl ank , SW Jordan : Evidence from apatite fi ssion track thermochronometry. *Tectonics*. **32**, 1513–1528 (2013).

124. W. Ghebreab, A. Carter, A. J. Hurford, L. Jouniaux, Constraints for timing of extensional tectonics in the western margin of the Red Sea in Eritrea. *Earth Planet. Sci. Lett.* **200**, 107–119 (2002).

125. B. P. Kohn, M. Eyal, History of uplift of the crystalline basement of Sinai and its relation to opening of the Red Sea as revealed by fission track dating of apatites. *Earth Planet. Sci. Lett.* **52**, 129–141 (1981).
